# Supplementary material for: Mapping the proteogenomic landscape enables prediction of drug response in acute myeloid leukemia
Source: Cell Rep Med. 2024 Jan 16;5(1):101359. doi: 10.1016/j.xcrm.2023.101359 (PMC10829797; doi:10.1016/j.xcrm.2023.101359)
Supplement: Document S1. Figures S1‒S5 [file mmc1.pdf]

Cell Reports Medicine, Volume *5*

Supplemental information

Mapping the proteogenomic landscape enables prediction of drug response in acute myeloid leukemia

James C. Pino, Camilo Posso, Sunil K. Joshi, Michael Nestor, Jamie Moon, Joshua R. Hansen, Chelsea Hutchinson-Bunch, Marina A. Gritsenko, Karl K. Weitz, Kevin Watanabe-Smith, Nicola Long, Jason E. McDermott, Brian J. Druker, Tao Liu, Jeffrey W. Tyner, Anupriya Agarwal, Elie Traer, Paul D. Piehowski, Cristina E. Tognon, Karin D. Rodland, and Sara J.C. Gosline

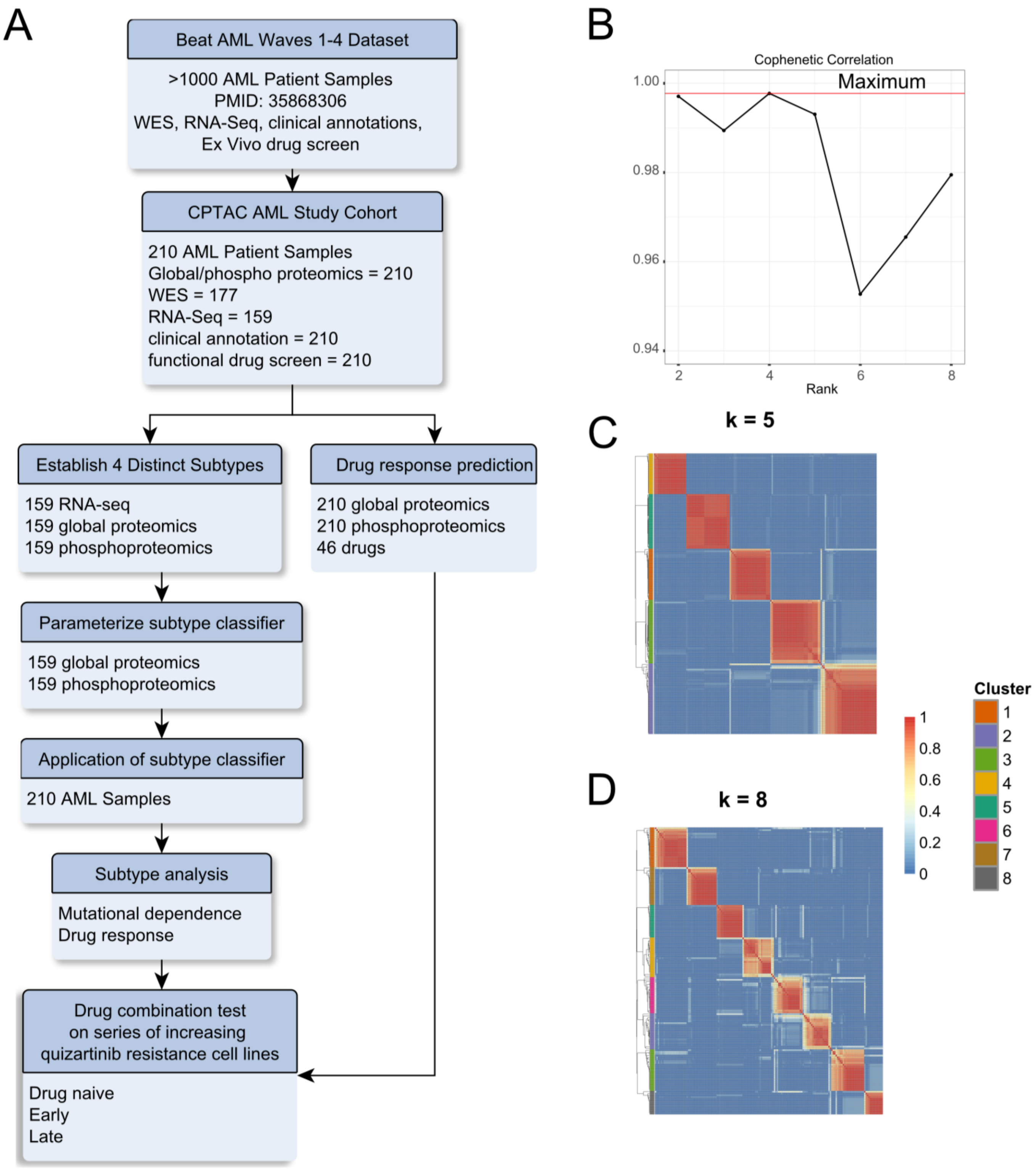

Figure S1. Patient cohort analysis. Related to Figure 1. (A) Flow of patient samples across modeling process. (B) The cophenetic correlation for each rank from NMF. For each rank k, this is a measure of the collective agreement between the clusters across 50 random initializations of NMF. Consensus heatmap of 50 NMF runs with k = 5 (C) and k = 8 (D) clusters. Ward method with Euclidian distance clustering was performed. The 159 samples are seen in the rows and columns, while the heatmap shows the proportion of runs in which samples were clustered together.

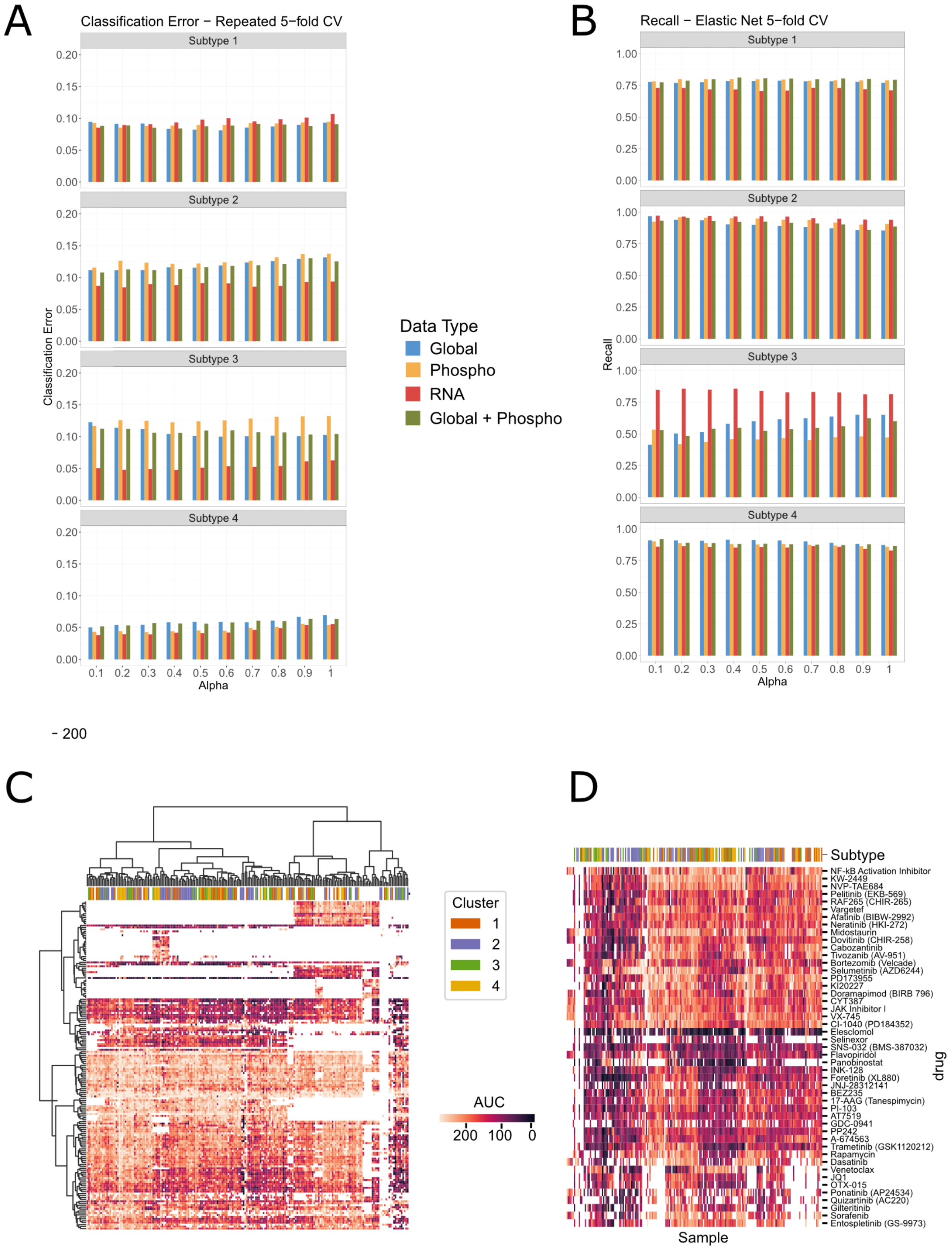

Figure S2. **Elastic net performance and extension.** Related to Figure 2. Classification error **(A)** and recall **(B)**, per subtype, for across values of α. **(C)** Heatmap of AUC values for available in the beatAML dataset **(D)** Resulting heatmap after filtering to include a minimum of 100 samples per drug.

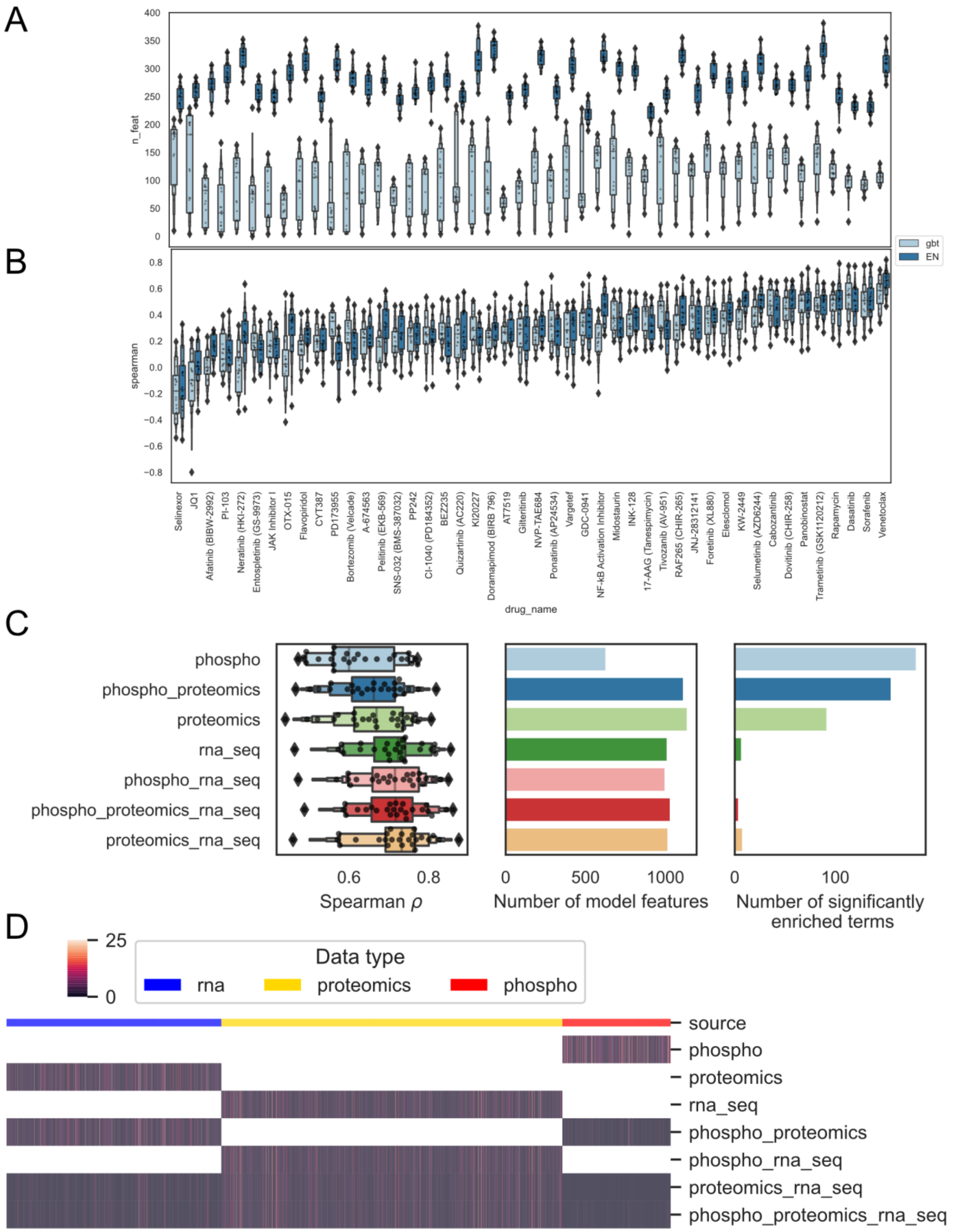

Figure S3. Predictive modeling of drug response using multi-omics. Related to STAR Methods. Number of features **(A)** and model performance **(B)** for Elastic Net (EN) or lightGBM (gbt). **(C)** Venetoclax performance for each data type and combinations of data type, as well as the number of features found, and the number of enriched terms for each data type. **(D)** Reoccurrence of model features for each data type. Since there were 5-repeat, 5-fold cross validation, a total of 25 models were constructed per data type. Thus, a feature could be selected 25 times. We see features are reselected across single and combination of data types, showing that additional data increases non-redundant information.

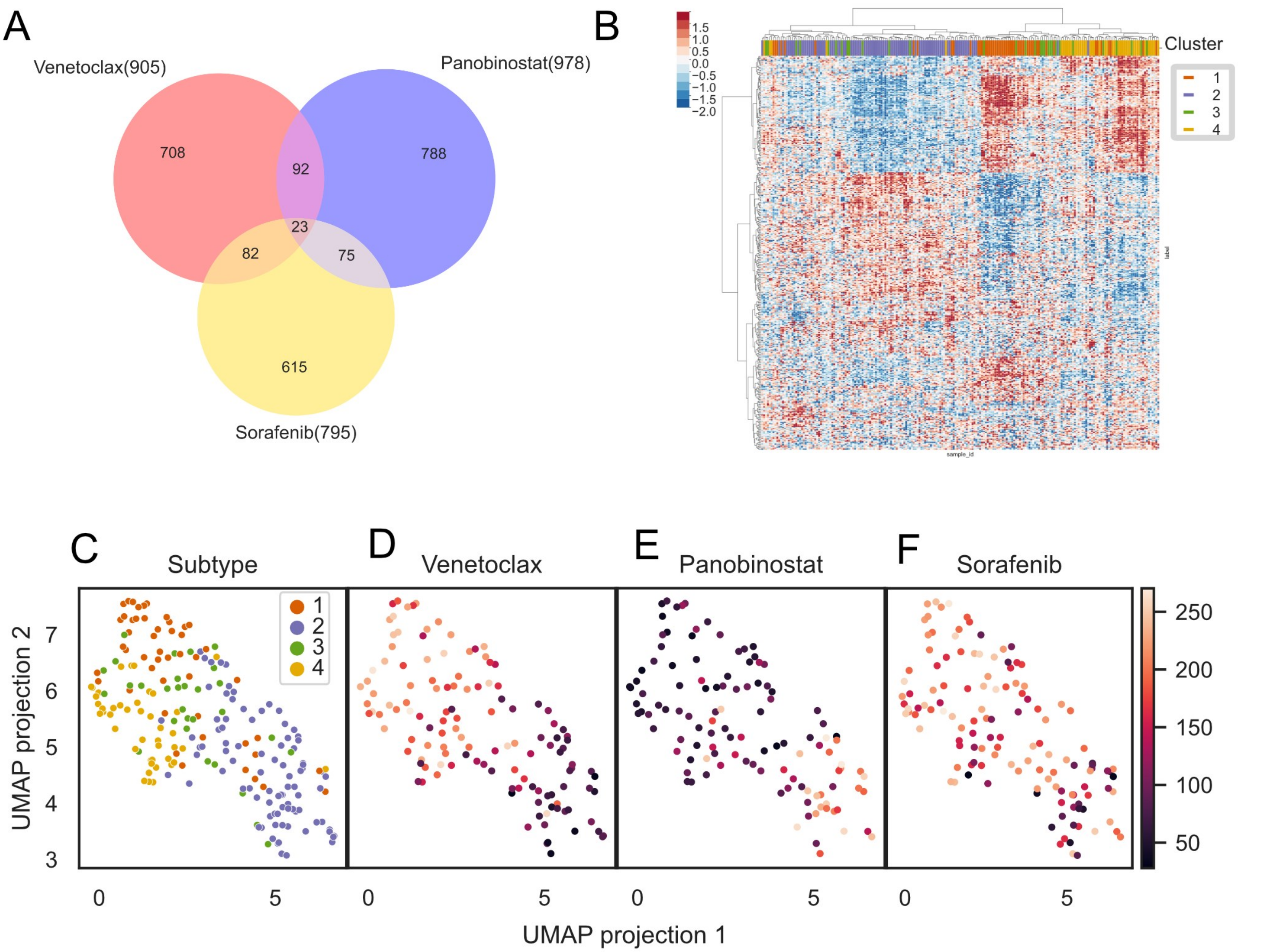

Figure S4. Feature comparisons between drugs of interest. Related to Figure 5. **(A)** Comparisons of features extracted using lightGBM. **(B)** Heatmap of the features from **(A)**, with unsupervised clustering. UMAP projection of the union of the features for each of the models, projected into two-dimensional landscape **(A-F)**. Color represented subtype **(C)** or AUC drug response (lower/darker values equates to more sensitive) **(D-E)**.

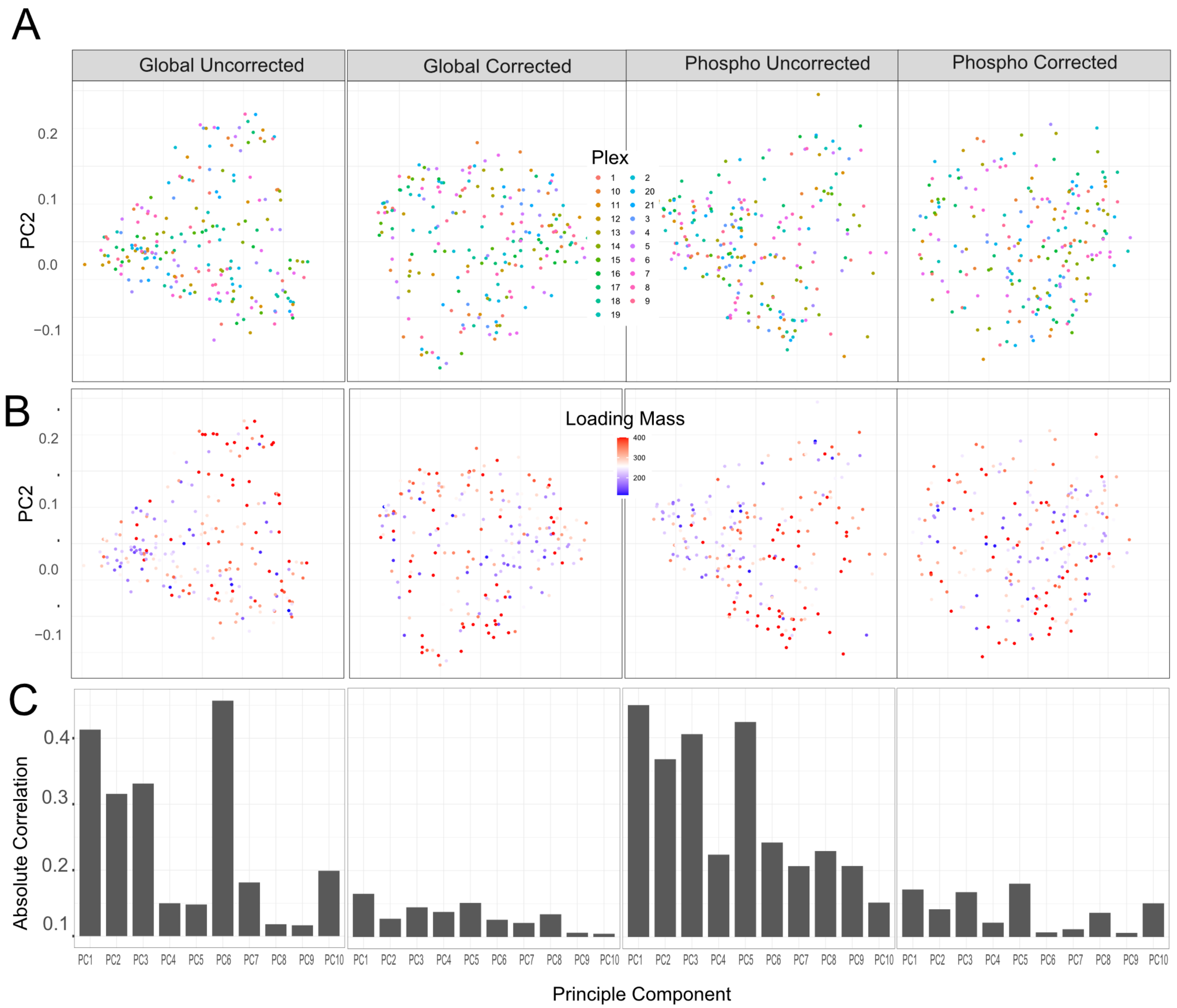

Figure S5. Batch correction of proteomics data. Related to STAR methods. First two principal components of Global and Phospho datasets before and after correction for Loading Mass **(A)** or Plex **(B)**. We found Loading Mass had a significant effect in 99% (global) and 93% (phospho) of the features while Plex had a significant effect in 14% (global) and 56% (phospho). After correction, neither Loading Mass nor plex no longer had any significant effect. The batch effect was assessed using BH-adjusted p-values from an F-test computed with the Limma package. **(C)** Correlation between Loading Mass and the first 10 principal components within Global and Phospho. Loading Mass has considerably higher correlation with the principal components in uncorrected data vs corrected data. After correction, little to no correlation is left between Loading Mass and the principal components.
