# Supplementary material for: Mapping the proteogenomic landscape enables prediction of drug response in acute myeloid leukemia
Source: Cell Rep Med. 2024 Jan 16;5(1):101359. doi: 10.1016/j.xcrm.2023.101359 (PMC10829797; doi:10.1016/j.xcrm.2023.101359)
Supplement: Document S2. Article plus supplemental information [file mmc8.pdf]

Cell Reports
Medicine

Article

Mapping the proteogenomic landscape enables prediction of drug response in acute myeloid leukemia

Graphical abstract

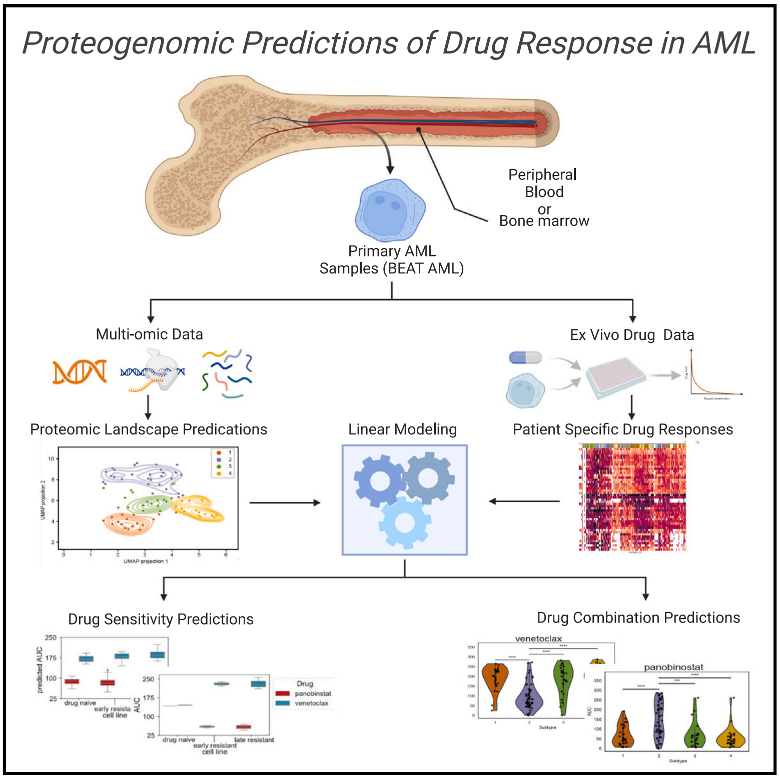

Highlights

- Proteogenomic characterization paired with drug response of 210 patients with AML
- Identification of 4 subtypes that correspond to AML drug response landscape
- Predictive drug response models for 46 drugs with venetoclax as a top performer
- Found that drug exposure changes cancer subtypes and alters drug sensitivity

Authors

James C. Pino, Camilo Posso, Sunil K. Joshi, ..., Cristina E. Tognon, Karin D. Rodland, Sara J.C. Gosline

Correspondence

rodland@ohsu.edu (K.D.R.),
sara.gosline@pnnl.gov (S.J.C.G.)

In brief

Pino et al. measure global proteomics and phosphoproteomics of 210 patients with acute myeloid leukemia (AML). Paired with genomic, transcriptomic, and *ex vivo* drug response data, they find 4 subtypes that correspond with distinct drug response profiles. Their models allow ideal treatment selection and provide optimized secondary treatment to relapsed patients.

Pino et al., 2024, Cell Reports Medicine *5*, 101359
January 16, 2024 © 2023 The Authors.
https://doi.org/10.1016/j.xcrm.2023.101359

Cell Reports Medicine

CellPress OPEN ACCESS

Article

Mapping the proteogenomic landscape enables prediction of drug response in acute myeloid leukemia

James C. Pino,[1] Camilo Posso,[1] Sunil K. Joshi,[2,3] Michael Nestor,[1] Jamie Moon,[1] Joshua R. Hansen,[1] Chelsea Hutchinson-Bunch,[1] Marina A. Gritsenko,[1] Karl K. Weitz,[1] Kevin Watanabe-Smith,[4] Nicola Long,[4] Jason E. McDermott,[1,5] Brian J. Druker,[2,3,4] Tao Liu,[1] Jeffrey W. Tyner,[2,3,6] Anupriya Agarwal,[2,3,4,6] Elie Traer,[2,3] Paul D. Piehowski,[1] Cristina E. Tognon,[2,3] Karin D. Rodland,[1,6,*] and Sara J.C. Gosline[1,7,*]

[1]Earth and Biological Sciences Directorate, Pacific Northwest National Laboratory, Richland, WA, USA
[2]Knight Cancer Institute, Oregon Health & Science University, Portland, OR, USA
[3]Division of Hematology & Medical Oncology, Department of Medicine, Oregon Health & Science University, Portland, OR, USA
[4]Division of Oncological Sciences, Oregon Health & Science University, Portland, OR, USA
[5]Department of Molecular Microbiology and Immunology, Oregon Health & Science University, Portland, OR, USA
[6]Department of Cell, Developmental, and Cancer Biology, Oregon Health & Science University, Portland, OR, USA
[7]Lead contact
*Correspondence: rodland@ohsu.edu (K.D.R.), sara.gosline@pnnl.gov (S.J.C.G.)
https://doi.org/10.1016/j.xcrm.2023.101359

SUMMARY

Acute myeloid leukemia is a poor-prognosis cancer commonly stratified by genetic aberrations, but these mutations are often heterogeneous and fail to consistently predict therapeutic response. Here, we combine transcriptomic, proteomic, and phosphoproteomic datasets with *ex vivo* drug sensitivity data to help understand the underlying pathophysiology of AML beyond mutations. We measure the proteome and phosphoproteome of 210 patients and combine them with genomic and transcriptomic measurements to identify four proteogenomic subtypes that complement existing genetic subtypes. We build a predictor to classify samples into subtypes and map them to a "landscape" that identifies specific drug response patterns. We then build a drug response prediction model to identify drugs that target distinct subtypes and validate our findings on cell lines representing various stages of quizartinib resistance. Our results show how multiomics data together with drug sensitivity data can inform therapy stratification and drug combinations in AML.

INTRODUCTION

Acute myeloid leukemia (AML) is characterized by a maturation block within myeloid lineage cells, resulting in the accumulation of blasts within the peripheral blood and bone marrow. This reduces healthy blood cell formation, resulting in decreased numbers of granulocytes, platelets, and red blood cells.[1] Although the number of Food and Drug Administration (FDA)–approved treatments for AML has increased over the past 5 years, the prognosis remains poor, with an approximate 5-year survival rate of 25% for individuals over the age of 20.[2] Targeted agents, such as those that inhibit the mutated gene FLT3, initially demonstrated promise in mutationally defined subsets of patients, but drug response is often transient and a majority of patients relapse.[3,4]

Numerous studies have evaluated genomic and transcriptomic features of AML in attempts to assign diagnostic groupings and identify a combination of features that can map patients to a "molecular landscape" that stratifies clinical outcome through the integration of multidimensional datasets.[5–10] The Beat AML research program, which was among these early efforts, also aimed to improve drug selection by collecting large quantities of molecular data together with functional data from *ex vivo* small-molecule inhibitor assays performed on freshly isolated patient leukemia cells.[11,12] Patient genomics and transcriptomics, as well as extensive clinical annotation, were paired with *ex vivo* drug response.[11,12] This dataset uncovered numerous genetic, transcriptomic, and microenvironmental drivers of AML pathogenesis and drug resistance,[13–17] and has been leveraged broadly in the field to examine the molecular features of drug response and clinical outcome.[18–23]

Although AML has been studied predominantly at the genetic level, advances in high-throughput technologies provide a complementary approach to investigate the disease at the proteomic level. Studies in solid tumors have demonstrated that proteomic analyses, including measurements of global protein levels and specific phosphosites, can better identify clinically relevant diagnostic and predictive patterns compared to transcriptomics or genomics alone.[24–27] This motivated the National Cancer Institute (NCI) to create the Clinical Proteomic Tumor Analysis Consortium (CPTAC), in which patient-derived samples were assayed using state-of-the-art mass spectrometry (MS) pipelines to produce proteomic and phosphoproteomic measurements of hundreds of tumors in breast, ovary, kidney, head and neck, endometrium, brain, and other tissues.[28–35] Efforts to study the

This is an open access article under the CC BY license (http://creativecommons.org/licenses/by/4.0/).

impact of the proteomic signatures on drug response have been previously evaluated in AML cell lines[24,36,37] and in primary AML patient samples.[18,36,38–40] In each study, these proteomic measurements demonstrated patterns that were not evident at the genomic or transcriptomic level.[18,41] Recent studies have shown that the use of proteomic signatures can also improve modeling of *ex vivo* drug response,[38,42] providing impetus for the proteomic characterization of a larger cohort to further map the molecular diversity of AML in response to drug treatment.

In this study, we explored the extent to which the molecular landscape, determined by genomic, transcriptomic, and proteomic measurements, can provide insight into *ex vivo* drug response. We applied our in-depth global and phosphoproteomic pipeline, developed under the NCI CPTAC program,[34,43,44] to a subset of 210 patients within the Beat AML cohort, which had been previously characterized via whole-exome sequencing, RNA sequencing, and *ex vivo* drug sensitivity measurements of 145 small-molecule inhibitors.[11,12] We integrated the transcriptomics, proteomics, and phosphoproteomics to identify four distinct proteogenomic subtypes of patients using non-negative matrix factorization. We then built a predictor of these subtypes to enable comparison with standard mutational profiling and cell line analysis. By mapping drug response profiles to our proteogenomic subtypes, we identified complementary drug profiles that suggested improved efficacy of specific drug combinations. We were also able to leverage the subtype predictor together with targeted machine learning to study acquired resistance to quizartinib, a drug used to treat FLT3-ITD (internal tandem duplication) AML patients that is currently undergoing FDA approval.[45] We predicted and experimentally validated a switch in the sensitivities of quizartinib-resistant cell lines to other drugs, depending on their proteogenomic state. In conclusion, we introduce a rich proteogenomic dataset that provides a resource for AML research and therapy stratification.

RESULTS

Our approach uses proteomic and phosphoproteomic characterization and analysis on a cohort of 210 patient samples. As shown in Figure S1A, we leverage 159 samples to identify subtypes that leverage proteomic, phosphoproteomic, and transcriptomic measurements. We then build a proteomic and phosphoproteomic–only predictor from these subtype assignments that allows us to classify the remaining patient samples, previously published cell lines, and, ultimately, new samples.

Multiomic clustering enables the definition of four distinct AML molecular subtypes

To evaluate the effect of clustering on a large population of patients, we selected 210 patient samples (Table S1) from the larger Beat AML cohort[11,12] based on the availability of samples with informed consent for research purposes and with an emphasis on FLT3-ITD mutations, given the advent of multiple new therapeutics targeting FLT3 mutations. From these samples, we generated proteomics and phosphoproteomics data, using MS-GF+ for peptide identification and tandem mass tags (TMT)–based isobaric labeling for accurate quantification (STAR Methods). We measured 401,999 peptides, comprising a total of 9,412 unique proteins, along with 94,789 phosphorylated peptides (phosphopeptides) belonging to 7,365 unique proteins, both with a false discovery rate (FDR) below 1%. We then removed features with over 50% of their intensities missing, yielding 8,521 proteins and 18,098 phosphosites (see STAR Methods). We removed batch effects due to continuous loading mass and plex differences using a Bayesian approach[19,34] (STAR Methods). This yielded 2 datasets, with a total of 7,084 proteins in the corrected global data, and 14,084 phosphosites in the corrected phosphoproteomics data.

We harmonized the global and phosphoproteomics data (n = 210) with the corresponding genomic (n = 177) and transcriptomic (n = 159) datasets from the Beat AML patient cohort, all of which possessed *ex vivo* drug response data[11,42] (Figure 1A; STAR Methods). To the best of our knowledge, this dataset represents the largest AML proteogenomic dataset with corresponding *ex vivo* screening data. To assign sample subtypes, we first focused on the unified dataset of 159 samples where mRNA, protein, and phosphosite measurements were obtained. We used non-negative matrix factorization[46] (NMF) to cluster the patients with all three data types into distinct groups (STAR Methods). Each grouping exhibited a different level of stability, so we varied the number of clusters from two to eight and measured the cophenetic correlation (Figures S1B–S1D) to determine that using four clusters resulted in the most reproducible clusters (Figure 1B). All data files are described in Table 1.

To examine the biological relevance of the clusters, we examined any relationship with mutation status and clinical annotations of the patient samples (Figure 1C). Cluster 1 was enriched with patients who had a prior diagnosis of myelodysplastic syndrome (MDS), mutations in the CSF3R gene, and mutations in the BCOR gene. Cluster 2 was enriched with relapsed patients, GATA2 mutations, WT1 mutations, and FLT3-ITD alterations. Cluster 3 contained samples with mutations in the FLT3 tyrosine kinase domain. Samples with mutated KRAS, NRAS, or NPM1 genes, as well as samples from newly diagnosed patients or taken from patients after chemotherapy treatment, were overrepresented among patients in cluster 4. We also examined the functional enrichment of the distinct transcripts, proteins, and phosphosites that were differentially expressed in each cluster (Table S2; STAR Methods) and found that the features were enriched in distinct biological terms (Figures 1D–1F). In the phosphosite analysis in Figure 1D, we found decreased PRKC kinase activity in cluster 2, and decreased CDK1 and MAPK kinase activity in cluster 3. In both the transcriptional and protein analyses, we found cluster 2 to be biologically distinct, with reduced immune signatures (signaling by interleukins, neutrophil degranulation, complement cascade) that were overrepresented in other clusters (Figures 1D–1F). We compared these clusters to other published AML subtypes and found that the transcriptional signature (Figure 1E) of cluster 2 captured signaling pathways (RHO GTPase, FCGR, and NFKB activation), whereas the protein signatures captured RNA splicing and processing and mitochondria-related signatures (Figure 1F), which aligns with Jayavelu et al.,[18] who observed enrichment of mitochondria-related signatures only at the protein level. This suggests that our cluster 2 is similar to their observed "c-mito" subtype. However, we wanted to explore the subtypes in the entire 210-patient cohort to further validate this suggestion.

CellPress
OPEN ACCESS

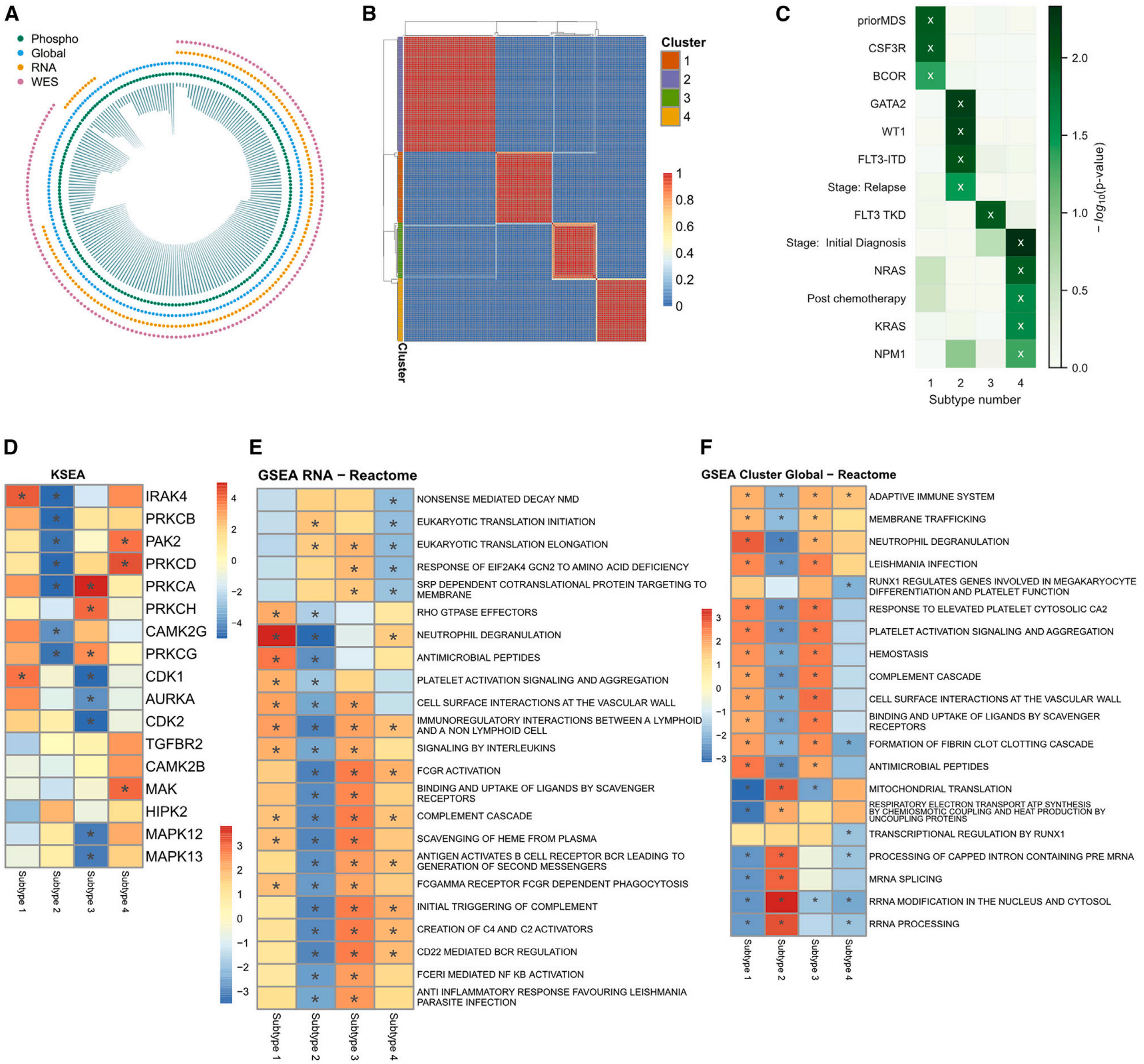

Figure 1. Multiomic clustering defines four biologically relevant AML subtypes

(A) Circos plot of data collected as part of this study. Bar height correlates with number of drugs assayed for each sample.

(B) Unsupervised multiomic clustering analysis combining mRNA, protein, and phosphosite measurements for a 159-patient cohort using NMF. Shading of squares indicates fraction of times patients were in the same cluster during randomization. Cluster annotations are located along the left-hand side.

(C) Cluster enrichment via Fisher's exact test, for gene-specific alterations and clinical variables, including "priorMDS," which indicates a prior diagnosis of MDS; "Stage," which indicates whether the sample was collected at initial diagnosis or at relapse; and "Post chemotherapy," which indicates the patient had received treatment. The x marks $p < 0.05$.

(D–F) Overrepresentation analysis using kinase-substrate enrichment analysis (KSEA) (D) and gene set enrichment analysis (GSEA) for RNA (E) and global proteomics (F), where * indicates adjusted $p < 0.05$.

Proteomic signatures of subtypes enable the classification of 210 patients and cell lines

To enable the subtypes defined from the NMF clustering to generalize across other datasets including cell lines and the 51 patients that were not classified above due to lack of mRNA measurements, we used a machine learning classifier to predict subtype from protein and phosphosite data (Figure S1). We evaluated a series of elastic net classification models[47] across combinations of mRNA, protein, and phosphosite data (see STAR Methods) to identify which data modality (or combination thereof) was best able to recreate the four subtypes. Using k-fold cross-validation to parameterize the model and evaluate it on held-out data, we

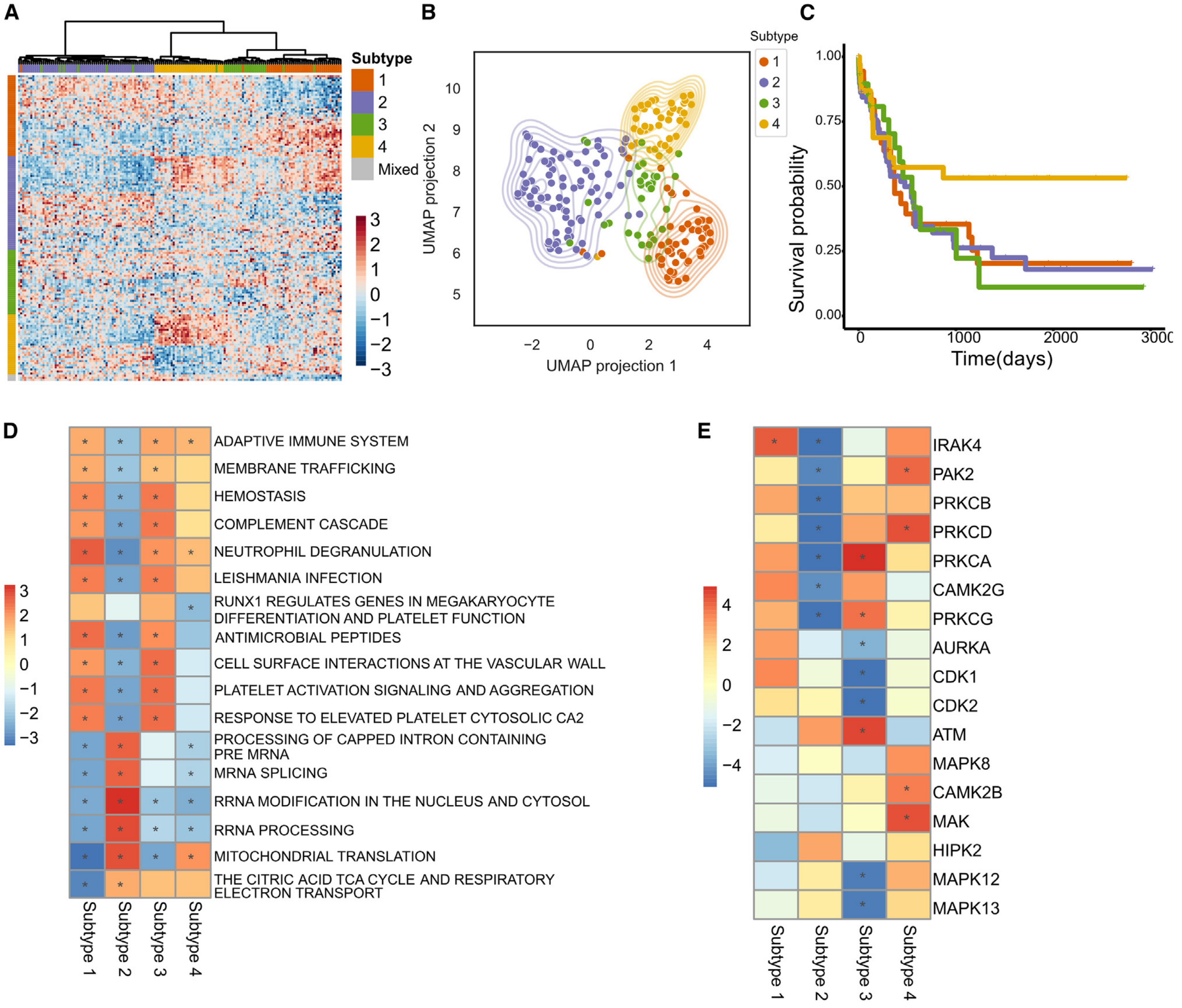

Figure 2. Subtype prediction expands classification to 210-patient cohort

(A) Expression of the 147 features used to classify patients according to subtype (rows) and the expression for each patient (columns). Resulting classification is annotated across the top. Row color corresponds to source of feature, with the gray color showing features found to be predictive of multiple subtypes.

(B) UMAP projections demonstrating how the features in (A) map patients into a two-dimensional landscape.

(C) Kaplan-Meier plots of patients according to subtype classification.

(D and E) Heatmap of enrichment scores for GSEA (D) and KSEA (E) results for each subtype using our full 210-patient proteomic or phosphoproteomic dataset. Color represents enrichment score; asterisk denotes an adjusted $p < 0.05$.

found that proteomics-only data (Figures S2A and S2B) was able to recapitulate the four subtypes, opening the possibility that patients could be subtyped based solely on proteomics data, without transcriptomics. However, we were not able to test this directly because no independent patient set was available. The optimized predictor used 147 proteins and phosphosites (Figure 2A; Table S3). The features of the model were not enriched in any biological process terms, likely due to the way elastic net aims to minimize the number of features, thereby preventing many overlapping, similar features from being included in the model. We then projected the 147 features across the 210 samples into a 2-dimensional landscape via uniform manifold approximation and projection (UMAP)[48] projection (Figure 2B) to confirm that the 147 features were sufficiently able to cluster patients according to subtype.

Using this proteome-only classification model, we were able to extend our subtype analysis to all 210 patient samples, including the 51 patients without RNA sequencing. By using the larger 210 patients, we found a statistically significant survival advantage among patients in subtype 4 ($p < 0.05$; Figure 2C). We also repeated our enrichment analysis from above that compared proteins and phosphosites that were enriched

CellPress
OPEN ACCESS

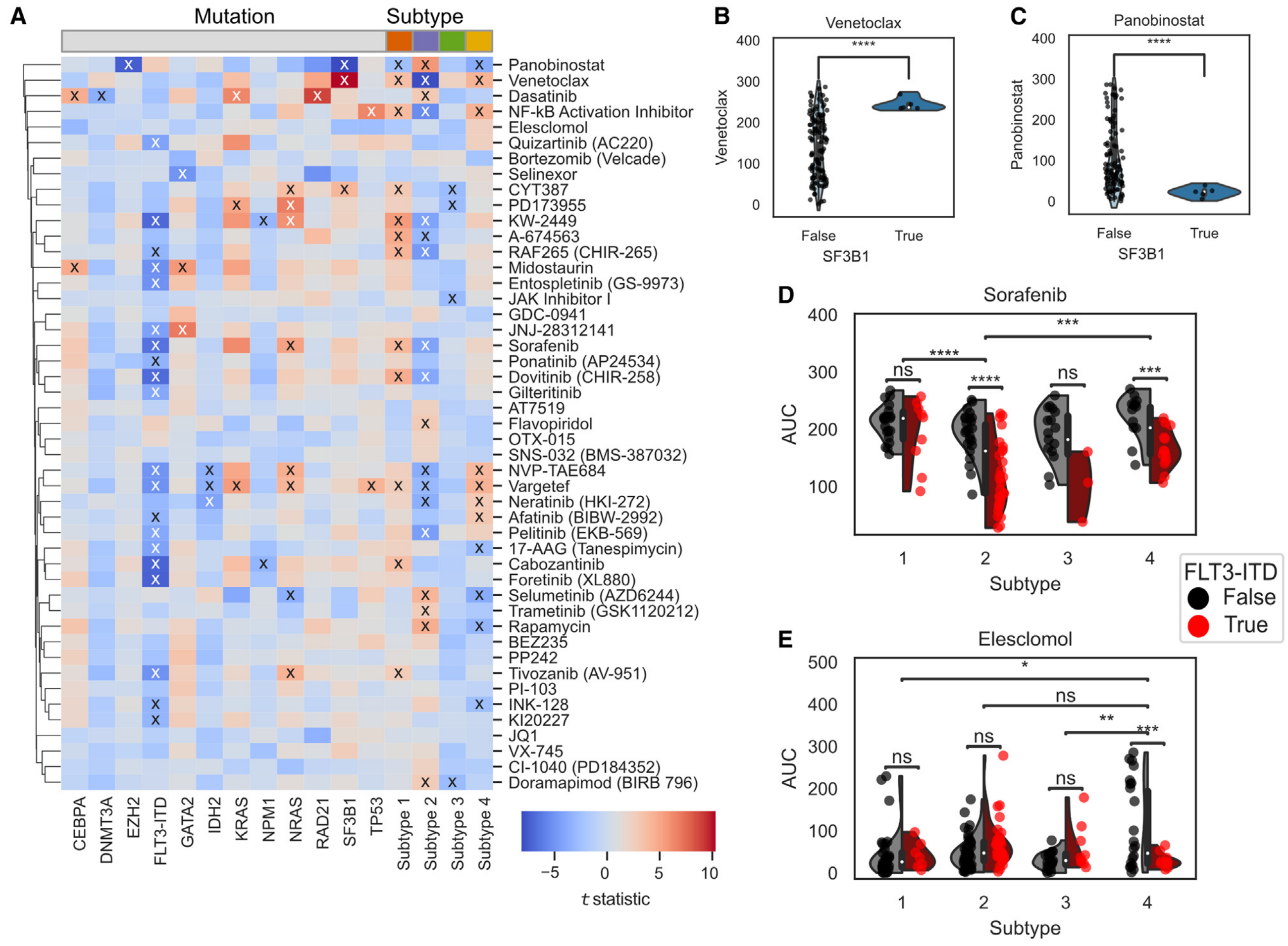

Figure 3. Mutational subtypes and proteomic subtypes stratify patient response of nonoverlapping sets of drugs

(A) Association of mutation status or subtype with drug response via Welch's paired t test. Shading represents t-statistic (legend inset). The x indicates adjusted $p < 0.05$.

(B and C) SF3B1 mutation status effect on response to venetoclax (B) or panobinostat (C).

(D and E) Three drug response profiles assessed by subtype (x axis) and FLT3-ITD status (color). Each * indicates significance level (e.g., *p = 0.01, **p = 0.001).

between samples of each predicted subtype. Specifically, subtype 2 is enriched in mitochondrial translation and the TCA cycle (Figure 2D), confirming that our subtype 2 maps to the mitochondrial distinct c-mito subtype described by Jayavelu et al.[18] Subtypes 1 and 3 share a similar protein signature (Figure 2D), but differ in kinase enrichment (Figure 2E), in which only IRAK4 appears enriched in subtype 1, whereas multiple kinases, including AURKA, a known regulator of AML therapy response,[23] are enriched in subtype 3. Thus, the biological content of the predicted subtypes is preserved from those determined from the multiomic clusters, further supporting the use of proteome-based classification to define the subtypes.

Proteomic subtypes complement mutational subtypes to improve drug response stratification

Given the wealth of *ex vivo* drug sensitivity data that were available through the analysis of the Beat AML cohort,[11,12] we sought to identify patterns between our multiomic subtypes and drug response. We obtained drug dose-response data for the 210 patient samples for up to 145 drugs from the Beat AML dataset,[11,12] using area under the curve (AUC) as a metric for response (Table S4; STAR Methods,). These values range from 1 to 300, with high values indicating more resistant samples and lower values corresponding to more sensitive samples. Measurements were not collected for all drugs across all patients, resulting in sparseness in the matrix (Figure S2C). To address this, we only considered drugs that were tested on a minimum of 100 patient samples and which had at least 10 patients considered to be "sensitive" according to the BeatAML criteria (AUC <100). The AUC values for the resulting 46 drugs (Table S3) are depicted in Figure S2D. We found that, using hierarchical clustering (Ward method), the drug response values for each sample did not cluster the patients according to each subtype (Figure S2D).

We tested whether knowing subtype and/or mutation could distinguish patient response across the 46 drugs in the *ex vivo* drug response dataset included in this study (Table S4). We used Welch's t test to evaluate the statistical association between each individual mutation and each subtype, shown in Figure 3A.

Using an FDR-corrected p value of 0.05, we found numerous associations (indicated by an "x" in Figure 3A). We were not surprised to find that FLT3-ITD samples displayed sensitivity in response for 20 of 46 drugs, including multiple FLT3 inhibitors such as sorafenib, KW-2449, midostaurin, dovitinib, quizartinib, and gilteritinib. Similarly, NRAS, KRAS, and TP53 mutations generally resulted in higher AUC values because these mutations generally make the tumor less responsive to all drugs. Interestingly, molecular subtype corresponded to drug sensitivity for 26 of the 46 drugs. Subtype 1 was correlated with many increases in resistance but only one increase in sensitivity, to the drug panobinostat. Subtype 2 shared many sensitivities to FLT3-ITD samples, accurately capturing the FLT3-ITD enrichment seen across those samples (Figure 1C). Interestingly, subtype 2 showed sensitivity to venetoclax (BCL2 inhibitor) and an NF-κB inhibitor, both not significantly different in FLT3-ITD samples, demonstrating that the multiomic subtypes captured distinct information about the molecular landscape compared to mutations. We also examined a strong difference in the SF3B1 mutated samples (Figures 3B and 3C) that showed a similar trend in sensitivity to panobinostat and resistance to venetoclax, seen in subtypes 1 and 4, and the opposite trend to subtype 2. These results suggest that SF3B1 is a key mediator of sensitivity to these drugs.

We then examined cases in which the combination of FLT3-ITD (the most common mutation in the cohort) status together with proteogenomic subtype were significant predictors of drug response using the same statistical test described above. By looking for statistical interactions between subtype and mutation, we found that the presence of an FLT3-ITD mutation only affected sorafenib drug response in patients assigned to subtype 2 (Figure 3D). We also found that most samples were sensitive to elesclomol (a drug that targets mitochondrial metabolism) across all of the samples, except those who were in subtype 4 and FLT3-WT (Figure 3E). Thus, AML proteogenomic subtypes and mutation status each capture distinct information that can be used for drug stratification, and in some cases, knowing both mutation status and subtype leads to an even greater ability to predict drug response.

Complementary behavior of drug combinations is only partially explained by drug target networks

We next leveraged the proteogenomic subtypes in combination with drug response to examine pairs of drugs that seemed to affect disparate sets of patients with the hypothesis that drugs with complementary profiles could be used in combination to prevent the outgrowth of subclones resistant to the initial targeted therapy. Toward this end, we mined the drugs from Figure 3A to select drugs that exhibited divergent responses—those whose responses targeted distinct subtypes of patient samples. We identified three such drugs: venetoclax, panobinostat, and sorafenib (Figures 4A–4C). Here, we found patients in subtype 2 to be more sensitive to venetoclax and sorafenib and less sensitive to panobinostat. Given this divergent behavior, we hypothesized that combination treatment should result in an additive effect, in which drugs target each sensitive population independently of the other drugs. We tested this using both venetoclax and panobinostat (V/P) as well as sorafenib and panobinostat (S/P). Interestingly, we saw this to be true for the V/P combination (Figure 4D), but we observed an antagonist effect for the S/P combination (Figure 4E).

Given that the targets of these drugs are known, we performed a simple visualization that leveraged knowledge of the affected signaling pathways using MAGINE[49] (see STAR Methods), building bidirectional paths between targets of each pair of drugs (Figures 4F and 4G). Although it is known that these drugs affect many different proteins, we were curious to see whether the underlying network structure between the drug targets were altered in a way that could explain the divergent drug response phenotypes. We looked at the shortest paths between BCL2 (venetoclax target) or FLT3 (one of the many targets of sorafenib) and HDAC1 (one of the histone deacetylases targeted by panobinostat). The results for V/P (Figure 4F) suggested that BCL2 and HDAC1 have a few short paths between the two targets and were therefore part of similar signaling networks. The S/P network, in Figure 4G, however, showed many more possible paths between HDAC1 and FLT3. This underscores the complexity of drug targeting in general and provides a plausible reason why the S/P combination did not have the desired effect. Ultimately, this analysis motivated us to look beyond the direct targets of venetoclax and panobinostat to identify protein signatures that predicted their response.

Distinct proteomic signatures are associated with response to V/P

To investigate the molecular mechanism that could explain the distinct nature of the V/P and S/P responses observed above, we used our machine learning-based approach[42] that uses linear modeling methods to select the combination of genomic, transcriptomic, and proteomic features that predict drug response (AUC). We have previously shown that linear models can be used to identify molecular markers of drug response in a 38-patient pilot study.[42] Therefore, we used this approach, with some additions (STAR Methods), to account for the expanded patient cohort of 210 samples and found that proteomic predictors could robustly predict drug response in 12 of 46 models that we evaluated (via correlation with data held out during cross-validation; Figure S3), including panobinostat and venetoclax.

We probed the signatures identified by the linear model to better understand the difference in drug combinations observed in Figure 4. The linear modeling approach identified 905 and 978 protein or phosphosite features that effectively modeled the AUC of venetoclax and panobinostat, respectively (Figure 5A; Table S5). The features (Figure 5A) and enriched Reactome pathways (Figure 5B) of these models were distinct. The predictive features of the sorafenib response shared little overlap with venetoclax (Figure S4A), despite exhibiting a similar response profile (Figure 4C). The panobinostat response was predicted by features representing the G1 phase of the cell cycle, DNA damage repair, and mitochondrial biogenesis, whereas the venetoclax response was predicted by features representing the G2/M phase of the cell cycle and programmed cell death.

We further explored the relationships between the minimal overlap of the two drug signatures (Figures 5A and 5B) and the complementary drug response (Figures 4A and 4B) to panobinostat and venetoclax. We clustered the patients based on the combining the proteins used in each of the models, resulting in

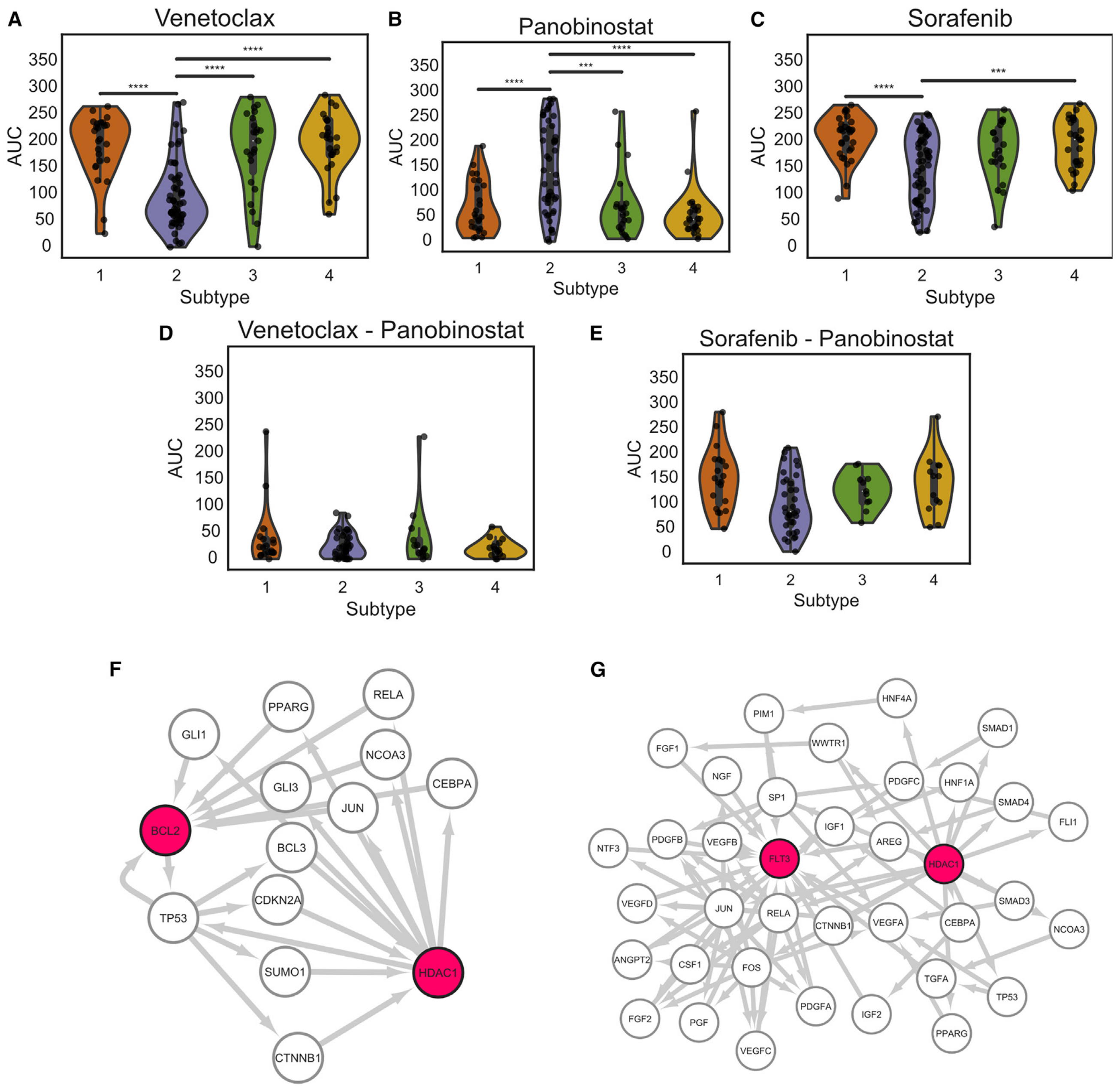

Figure 4. Distribution of *ex vivo* drug responses by subtype

(A–C) Distribution of AUC by subtype for (A) venetoclax, (B) panobinostat, and (C) sorafenib. The * indicates FDR-corrected significance of differences between classes according to Welch's t test p value; * = 1e−2, ** = 1e−3.

(D and E) Distribution of AUC values by subtype for the combination of (D) venetoclax and panobinostat and (E) sorafenib and panobinostat.

(F and G) Signaling networks created using the shortest paths between drug targets, using the MAGINE subnetwork tool. (F) Subnetwork between the primary target of venetoclax (BCL2) and a primary target of panobinostat (HDAC1). (G) Subnetwork between a primary target of sorafenib (FLT3) and a primary target of panobinostat (HDAC1).

1,611 protein expression features (Figure 5C) and then projected the features into a two-dimensional landscape using UMAP (Figure 5D). We found the location of a patient on the drug signature–imposed landscape to reflect the distinct patient responses to venetoclax and panobinostat (Figures 5E and 5F) and sorafenib (Figures S4C–S4F). This projection not only separated patients based on drug response but also grouped together subtypes, which is apparently an emergent property of the model. Thus, for the case of venetoclax and panobinostat, global proteomic and phosphoproteomic features used to predict drug response also contain information that relates directly to our independently determined subtypes. This suggests that the proteomics-based

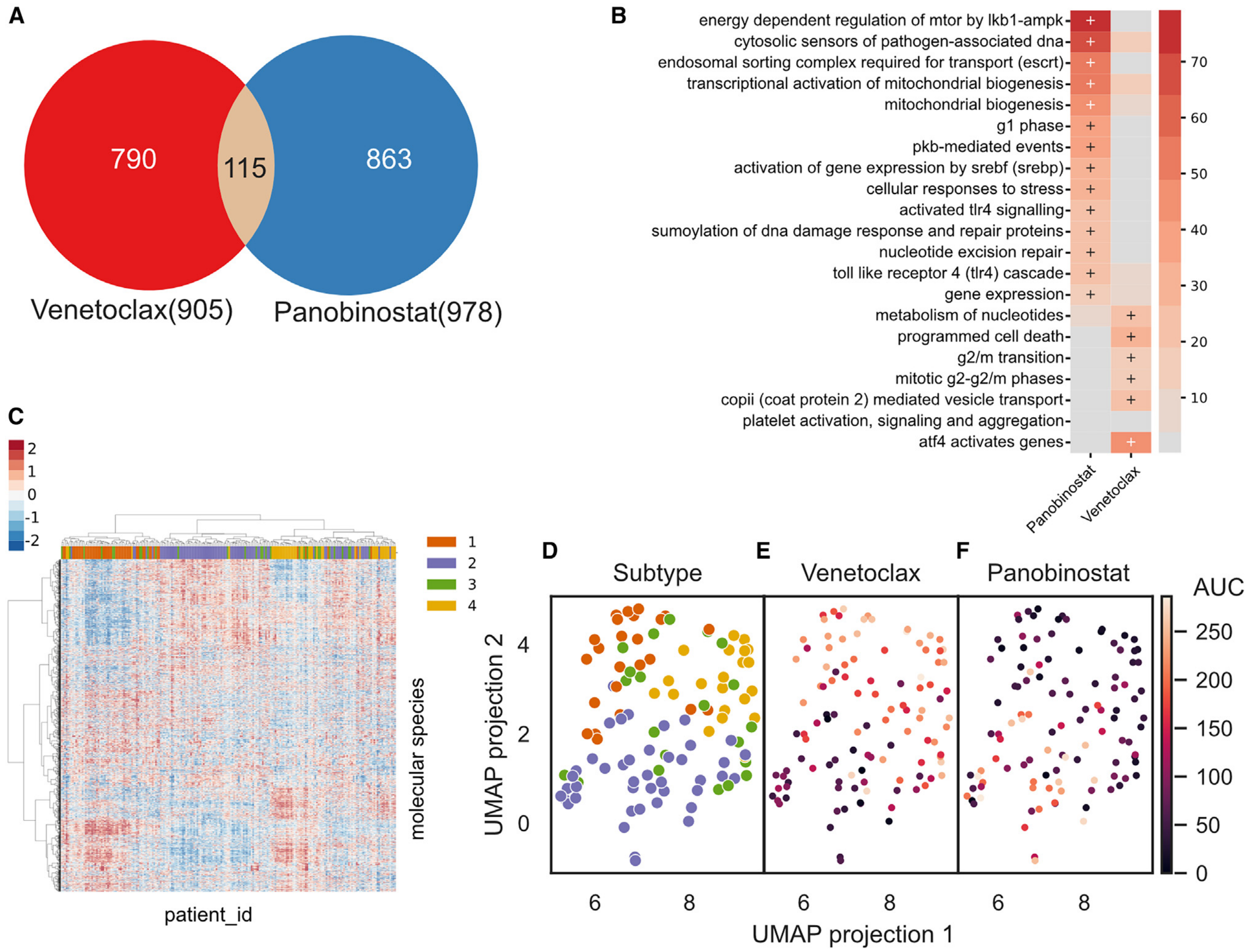

Figure 5. Feature analysis of drug prediction models

(A) Venn diagram showing the number of features in each drug model.

(B) Reactome terms overrepresented among the molecular features shown in (A). Colors represent the combined score from enrichR. + indicates significantly enriched terms based on adjusted $p < 0.05$.

(C) Unsupervised clustering of the molecular features extracted from the drug response models. Molecular features from AUC regression model.

(D) UMAP 2-dimensional projection of samples by the features extracted from the models, colored by subtype classification.

(E) Same UMAP representation as (D) but colored by venetoclax AUC.

(F) Same UMAP representation as (D) and (E) but colored by panobinostat AUC.

subtyping and predictive modeling could be used to predict V/P response in unseen samples.

Proteogenomic subtypes together with drug-specific signatures enable the prediction of drug response in AML

To evaluate how the landscape derived from the proteogenomic subtypes (Figure 2) could be leveraged together with the drug signature landscape (Figure 5) to predict drug response, we applied both models to previously published AML cell lines.[21] These MOLM-14 cell lines have FLT3-ITD and parental cells are sensitive to the FLT3 inhibitor quizartinib. However, after continuous exposure to quizartinib, MOLM14 cells can develop resistance via a two-stage process, an early resistance phase driven by changes in protein expression in response to the presence of stromal survival signals, followed by the emergence of mutations in late resistance that render survival ligand independent.[21,23] We asked whether the proteomic signatures measured at each stage captured their resistance profile and enabled prediction of the drug response.

Compared to the patient sample data, all MOLM14 samples clustered tightly in the UMAP space (Figure 6A), representing their overall homogeneity compared to patient samples. We then classified cells with evolving stages of resistance according to the proteogenomic subtypes (Figure 6B) and found that the parental, or drug-naive, cells fall within subtype 2, whereas the development of resistance to quizartinib caused the cells to shift to subtypes 1, 3, and 4, with subtype 3 dominating in late resistance (Figure 6B). Leveraging subtype response stratification (based on proteomics alone), we predicted that drug-naive and

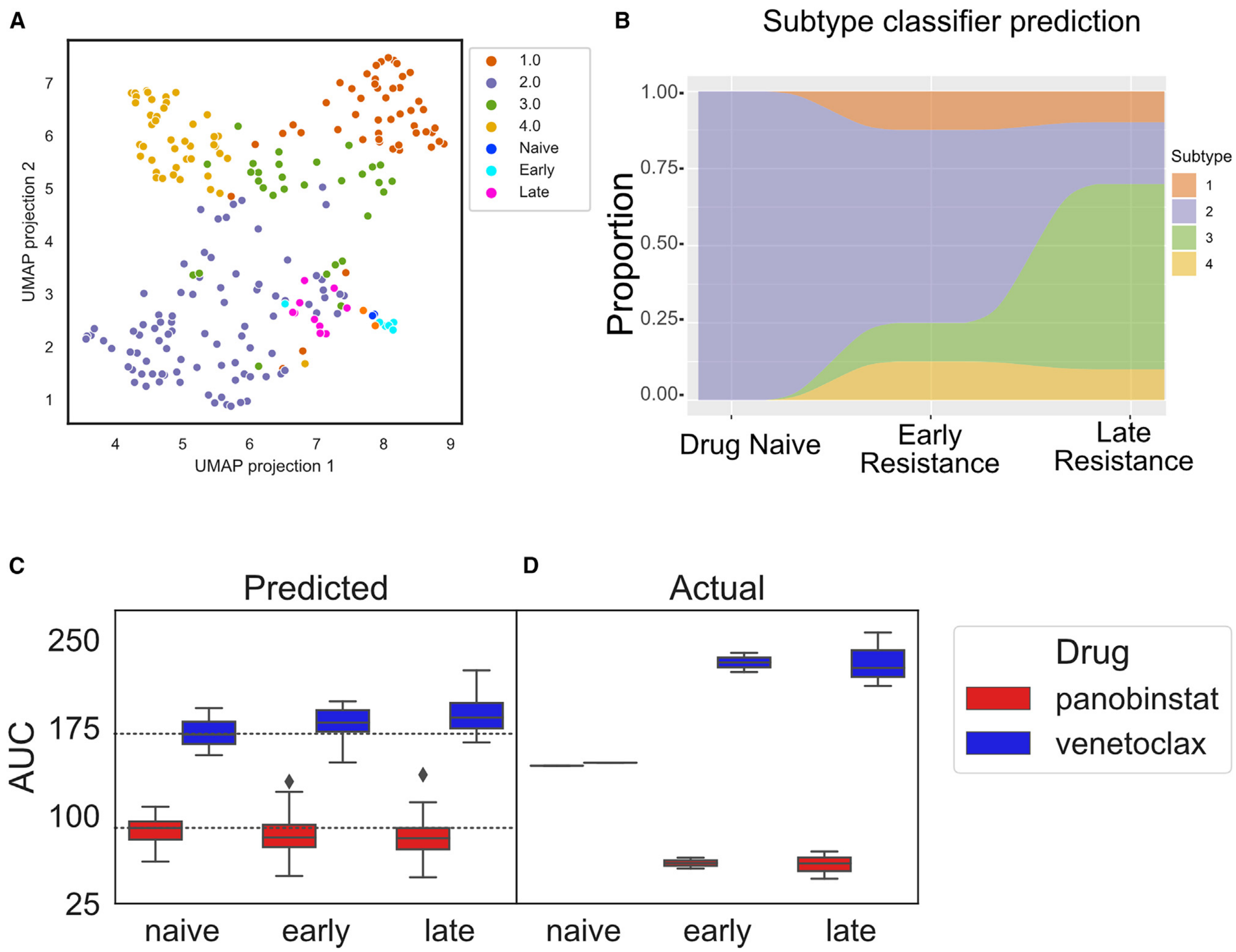

Figure 6. Predicting drug sensitivity following FLT3 inhibition induced landscape changes

(A) Proteogenomic subtype projection of patient samples together with MOLM14 naive, early resistant, and late resistant cell lines using UMAP.

(B) ElasticNet prediction of subtype of cell lines. Multiple measurements per cell stage allows a percentage projection of subtype.

(C) AUC predictions of cell lines across the different MOLM14 cell lines, with median AUC of naive cells marked by dashed line.

(D) Experimental results of venetoclax and panobinostat sensitivity on same cell lines; n = 4 for each condition.

early resistant cells would be more sensitive to subtype 2 drugs (venetoclax or sorafenib) and late resistant cells would be more sensitive to panobinostat (subtype 3 sensitive drug).

We then applied our venetoclax and panobinostat models to predict responses for all quizartinib naive, early, and late resistance samples. Shown in Figure 6C, the models predicted an increase in sensitivity to panobinostat and a decrease in sensitivity to venetoclax as cells became more resistant to quizartinib. We then validated these predictions by treating naive, early, or late quizartinib resistant cell lines with panobinostat or venetoclax in a 3-day drug sensitivity assay (see STAR Methods) and evaluated the effect of each drug on cell survival. As predicted, the MOLM14 cells became more resistance to venetoclax across early and late resistance stages, with a decrease in the proportion of the venetoclax-sensitive subtype 2 (Figure 6D). Panobinostat exhibited the opposite trend, in which the cells became more sensitive to panobinostat as the proportion of subtype 3 increased. These results provide *in vitro* validation of our models and showcase the value of proteomic subtyping to predict drug responses as tumors become resistant to a standard course of treatment.

DISCUSSION

Despite advances in the treatment of AML, its prognosis remains poor owing to its clinical and genetic heterogeneity. Many studies have shown that phenotype cannot be predicted solely by genotype,[50–52] and examination of mRNA expression does not fully capture the extent of signaling changes found within the leukemic proteome, which ultimately influences therapy response. Thus, the integration and aggregation of orthogonal multiomic datasets—genomics, transcriptomics, proteomics, and phosphoproteomics—with corresponding *ex vivo* pharmacologic screening holds the promise of bridging the gap in our understanding between the genotype and

Table 1. Location of processed proteomics files on synapse

Patients	Data type	File	Table
210	Proteomics	syn25714248	syn25808020
210	Phosphoproteomics	syn32528196	syn26477193
159	Transcriptomics	syn32529921	syn26545877
177	WES	syn32533104	syn26428827
210	Clinical annotations spreadsheet	syn25796769	syn52299924
210	All files (Synapse dataset)	syn52203674	

phenotype of leukemia cells. Such deep and quantitative characterization of the AML landscape may provide new diagnostic and mechanistic insights that may inform the development of new therapies.

In this study, we showcase the value of proteomics measurements as an additional approach to identifying potential therapies in AML. The proteogenomic subtypes presented in Figure 1 are linked to survival (Figure 2C) and underlying pathway activity (Figures 1D–1F, 2D, and 2E). Specifically, protein level measurements enabled confirmation of RNA-based pathway enrichment signatures and the addition of kinase-specific activity, in terms of potential targetable pathways such as CDKs, MAPKs, and PRKs (Figure 1D). These subtypes support findings reported in other AML cohorts, such as the increased phosphorylation of PRKCD observed in subtype 4, which was also identified in an independent AML proteomics cohort[53–55] and increased mitochondrial activity, oxidative phosphorylation, and poor overall survival in subtype 2, which aligns with the c-mito subtype identified by Jayavelu et al.[18]

Furthermore, this work, alongside other proteomic studies in AML,[38,56] suggests that proteomic signatures can provide a complementary approach for treatment selection in addition to the use of genomic features. We observed that some genetic features are associated with proteogenomic subtypes (Figure 1C)—for example, FLT3-ITD events were enriched in subtype 2 ($p = 0.01$), and NPM1 mutations were enriched in subtype 4 ($p = 0.04$). Of note, proteogenomic subtyping provides an opportunity to augment the use of genetic abnormalities to select targeted therapies. Our analysis of the multikinase inhibitor, sorafenib, provides a clear example (Figure 3D). By layering the proteomic subtypes with FLT3-ITD status, we observed that FLT3-ITD patients within subtypes 2 were more sensitive to sorafenib than corresponding FLT3-ITD^{+} patients in subtypes 1 and 4 (Figures 3D and 4C), demonstrating that proteomics can reveal additional biological insights that predict drug response beyond mutational status alone. Similarly, Casado et al. show that phosphoproteomic signatures predicted with greater accuracy responses to the FLT3 inhibitor midostaurin than the presence of FLT3 mutations alone.[38] This fits with clinical data from the SORAML trial,[57] which reported that FLT3 mutation status alone was not indicative of increased event-free survival with sorafenib treatment in conjunction with chemotherapy, likely due to the ability of sorafenib to target multiple signaling pathways and the signaling differences present in different subtypes of AML.

Cancers can adapt quickly to drug treatment through rapid changes in their overall proteogenomic landscape, and this form of early adaptation can support the development of mutation-based mechanisms of drug resistance.[23] Here, we found that by monitoring the proteomic state throughout treatment, we can track subtype transitions and predict responses to second-line therapies. Specifically, we show how quizartinib treatment can shift cells from being sensitive to the BCL2 inhibitor venetoclax to being more sensitive to the HDAC inhibitor panobinostat (Figure 6B). Quizartinib treatment effectively shifts the location of the cell lines across the landscape. Although drug-naive MOLM-14 cells are relatively sensitive to quizartinib, cells that adapt to quizartinib exposure alter their[58] protein signaling, possibly becoming more differentiated, and thus gaining resistance to venetoclax[59,60] and sensitivity to panobinostat.[12] the predictions, trained on patient samples, do not match the cell line validation results in scale, but they do match in direction and suggest a potential avenue by which drug combinations can be predicted/tested in other studies, which is prescient considering the number of current clinical trials targeting multiple AML targets, especially those using FLT3 inhibitors and venetoclax.[61–63] Our models could be used to predict initial treatments based on subtypes, as well as to predict responses to follow-up interventions, which can be used in designing optimal sequential applications[58] of treatments. Thus, frequent monitoring of patient samples throughout treatment may prove useful in maintaining responses to therapy in a dynamic disease.

The strength of this dataset lies in its ability to integrate drug sensitivity data with proteogenomic characterization of primary AML cells, enabling us to create a robust framework that can be used to predict specific drug response patterns from the proteogenomic landscape. Our work underscores the value of proteomic-based subtyping and how it can be leveraged to identify effective combinations of drugs for specific subsets of patients, bringing us closer to the goal of precision medicine. Machine learning offers an effective tool for synthesizing and analyzing the amassing omics data to extract prominent features that can be used to develop mechanistic models for hypothesis generation.[49,64] As a first step, we look forward to the harmonization of our dataset with other recently published proteomic studies in AML[18,23,36,37,39,40] to improve prognostication, and better understand the biology of drug response in AML patients.

Limitations of the study

The dynamic nature of AML, however, is one of the main limitations of this study. For example, the bulk processing of the samples cannot account for the known clonal heterogeneity of AML[65–69] and differences in overall balance between stromal and leukemic cells. Rapidly evolving improvements in mass spectrometry are making it possible to analyze patient samples after enriching for specific cell types based on cell surface determinants. In addition, the single-point time measurements fail to capture the dynamic response of the disease. This could be ameliorated by adding more time points to our drug study in the hope of better elucidating the mechanisms of resistance. Protocols featuring long-term culture of primary cells and longitudinal drug exposure[70,71] coupled with improved technologies

for the analysis of ultra-small samples will undoubtedly enable a more dynamic analysis of cellular responses. These types of technological improvements will help characterize drug response across AML studies. In addition, although our MOLM14 drug-resistant cell line models prove promising results, we are limited until we expand the findings in additional AML cell lines, AML mouse models, and, ultimately, in patient samples after treatment.

STAR★METHODS

Detailed methods are provided in the online version of this paper and include the following:

- KEY RESOURCES TABLE
- RESOURCE AVAILABILITY
  - Lead contact
  - Materials availability
  - Data and code availability
- EXPERIMENTAL MODEL AND STUDY PARTICIPANT DETAILS
  - Sample collection
  - Quizartinib resistant cell line generation
- METHOD DETAILS
  - *Ex vivo* drug screening analysis
  - Protein digestion and tandem mass tag (TMT) labeling
  - Phosphopeptide enrichment using IMAC
  - LC-MS/MS analysis
  - TMT global proteomics data processing
  - TMT phosphoproteomics data processing
  - Correcting proteomics and phosphoproteomics data for batch effect and loading mass
  - Multiomic clustering of patient subtypes via non-negative matrix factorization
  - Proteomic subtype predictor
  - Functional enrichment of subtypes
  - Prediction of drug-specific response
  - Signaling network construction
- QUANTIFICATION AND STATISTICAL ANALYSIS

SUPPLEMENTAL INFORMATION

Supplemental information can be found online at https://doi.org/10.1016/j.xcrm.2023.101359.

ACKNOWLEDGMENTS

We are extremely thankful to our patients for their time and precious tissue samples. Pacific Northwest National Laboratory is operated for the US Department of Energy by Battelle Memorial Institute under contract DE-AC05-76RL01830. This work was supported by the NCI's Office of Cancer Clinical Proteomics Research (CPTAC) under U01CA271412 awarded to P.D.P., J.W.T., B.J.D., and K.D.R.. S.K.J. is supported by the ARCS Scholar Foundation, The Paul & Daisy Soros Fellowship, and the NCI (F30CA239335). A.A. is supported by the American Cancer Society (RSG-17-187-01-LIB), the NCI (R01 CA229875-01A1), the National Heart, Lung, and Blood Institute (R01 HL155426-01), the Alex's Lemonade Stand Foundation/RUNX1 Research Program, and the Evans MDS Foundation.

AUTHOR CONTRIBUTIONS

Conceptualization: K.D.R., C.E.T., S.J.C.G., J.C.P., C.P., S.K.J., J.W.T., E.T., and A.A. Methodology: J.C.P., C.P., S.J.C.G., K.D.R., C.E.T., J.W.T., P.D.P., and B.J.D. Software: J.C.P., C.P., S.J.C.G., and J.M. Validation: J.C.P., C.P., S.K.J., K.W.-S., and S.J.C.G. Data curation: J.C.P., C.P., M.N., K.W.-S., and N.L. Formal analysis: J.C.P., C.P., and S.J.C.G. Investigation: P.D.P., M.A.G., J.R.H., C.H.-B., K.K.W., and S.K.J. Resources: C.E.T., M.A.G., C.H.-B., and J.R.H. Writing – original draft: J.C.P., C.P., and S.J.C.G. Writing – review & editing: J.C.P., C.P., S.J.C.G., C.E.T., S.K.J., K.D.R., J.E.M., T.L., J.W.T., A.A., E.T., P.D.P., and B.J.D. Visualization: J.C.P., C.P., and S.J.C.G. Supervision: K.D.R., P.D.P., J.E.M., S.J.C.G., and C.E.T. Project administration: K.D.R., S.J.C.G., and C.E.T. Funding acquisition: B.J.D., J.E.M., T.L., J.W.T., A.A., E.T., P.D.P., K.D.R., and S.J.C.G.

DECLARATION OF INTERESTS

J.W.T. has received research support from Agios, Aptose, Array, AstraZeneca, Constellation, Genentech, Gilead, Incyte, Janssen, Petra, Seattle Genetics, Syros, and Takeda and is on the Scientific Advisory Board of Recludix. C.E.T. has received support from Notable Labs. B.J.D. is/was on the Scientific Advisory Board of Adela Bio, Aileron Therapeutics (inactive), Celgene, Cepheid, DNA SEQ, Nemucore Medical Innovations, Novartis, RUNX1 Research Program, Therapy Architects/ALLCRON (inactive), and Vivid Biosciences (inactive); is/was on the Scientific Advisory Board of and owns/owned stock in Aptose Biosciences, Blueprint Medicines, Enliven Therapeutics, GRAIL, Iterion Therapeutics, and Recludix Pharma; is/was on the Board of Directors and owns/owned stock in Amgen and Vincerx Pharma; is/was on the Board of Directors of Burroughs Wellcome Fund, CureOne; is/was on the Joint Steering Committee of Beat AML (Leukemia & Lymphoma Society); is/was on the Advisory Committee of Multicancer Early Detection Consortium; is the Founder of VB Therapeutics; has/had sponsored research agreements with Enliven Therapeutics and Recludix Pharma; receives/received clinical trial funding from AstraZeneca and Novartis; receives/received royalties from Patent 6958335 (Novartis exclusive license), the Oregon Health & Science University, and the Dana-Farber Cancer Institute (one Merck exclusive license, one CytoImage, Inc. exclusive license, and one Sun Pharma Advanced Research Company nonexclusive license); and holds US Patents 4326534, 6958335, 7416873, 7592142, 10473667, 10664967, and 11049247.

INCLUSION AND DIVERSITY

We support inclusive, diverse, and equitable conduct of research.

Received: May 25, 2023
Revised: October 20, 2023
Accepted: December 10, 2023
Published: January 16, 2024

REFERENCES

1. Dong, Y., Shi, O., Zeng, Q., Lu, X., Wang, W., Li, Y., and Wang, Q. (2020). Leukemia incidence trends at the global, regional, and national level between 1990 and 2017. Exp. Hematol. Oncol. *9*, 14.
2. Key Statistics for Acute Myeloid Leukemia (AML). https://www.cancer.org/cancer/acute-myeloid-leukemia/about/key-statistics.html.
3. McMahon, C.M., Ferng, T., Canaani, J., Wang, E.S., Morrissette, J.J.D., Eastburn, D.J., Pellegrino, M., Durruthy-Durruthy, R., Watt, C.D., Asthana, S., et al. (2019). Clonal Selection with RAS Pathway Activation Mediates Secondary Clinical Resistance to Selective FLT3 Inhibition in Acute Myeloid Leukemia. Cancer Discov. *9*, 1050–1063.
4. Fletcher, L., Joshi, S.K., and Traer, E. (2020). Profile of Quizartinib for the Treatment of Adult Patients with Relapsed/Refractory FLT3-ITD-Positive Acute Myeloid Leukemia: Evidence to Date. Cancer Manag. Res. *12*, 151–163.

5. Cancer Genome Atlas Research Network; Ley, T.J., Miller, C., Ding, L., Raphael, B.J., Mungall, A.J., Robertson, A.G., Hoadley, K., Triche, T.J., Laird, P.W., et al. (2013). Genomic and epigenomic landscapes of adult de novo acute myeloid leukemia. N. Engl. J. Med. *368*, 2059–2074.

6. Patel, J.P., Gönen, M., Figueroa, M.E., Fernandez, H., Sun, Z., Racevskis, J., Van Vlierberghe, P., Dolgalev, I., Thomas, S., Aminova, O., et al. (2012). Prognostic relevance of integrated genetic profiling in acute myeloid leukemia. N. Engl. J. Med. *366*, 1079–1089.

7. Papaemmanuil, E., Gerstung, M., Bullinger, L., Gaidzik, V.I., Paschka, P., Roberts, N.D., Potter, N.E., Heuser, M., Thol, F., Bolli, N., et al. (2016). Genomic Classification and Prognosis in Acute Myeloid Leukemia. N. Engl. J. Med. *374*, 2209–2221.

8. Gentles, A.J., Plevritis, S.K., Majeti, R., and Alizadeh, A.A. (2010). Association of a leukemic stem cell gene expression signature with clinical outcomes in acute myeloid leukemia. JAMA *304*, 2706–2715.

9. Ng, S.W.K., Mitchell, A., Kennedy, J.A., Chen, W.C., McLeod, J., Ibrahimova, N., Arruda, A., Popescu, A., Gupta, V., Schimmer, A.D., et al. (2016). A 17-gene stemness score for rapid determination of risk in acute leukaemia. Nature *540*, 433–437.

10. Wang, Y.-H., Lin, C.-C., Yao, C.-Y., Hsu, C.-L., Hou, H.-A., Tsai, C.-H., Chou, W.-C., and Tien, H.-F. (2020). A 4-gene leukemic stem cell score can independently predict the prognosis of myelodysplastic syndrome patients. Blood Adv. *4*, 644–654.

11. Tyner, J.W., Tognon, C.E., Bottomly, D., Wilmot, B., Kurtz, S.E., Savage, S.L., Long, N., Schultz, A.R., Traer, E., Abel, M., et al. (2018). Functional genomic landscape of acute myeloid leukaemia. Nature *562*, 526–531.

12. Bottomly, D., Long, N., Schultz, A.R., Kurtz, S.E., Tognon, C.E., Johnson, K., Abel, M., Agarwal, A., Avaylon, S., Benton, E., et al. (2022). Integrative analysis of drug response and clinical outcome in acute myeloid leukemia. Cancer Cell *40*, 850–864.e9.

13. Nechiporuk, T., Kurtz, S.E., Nikolova, O., Liu, T., Jones, C.L., D'Alessandro, A., Culp-Hill, R., d'Almeida, A., Joshi, S.K., Rosenberg, M., et al. (2019). The TP53 Apoptotic Network Is a Primary Mediator of Resistance to BCL2 Inhibition in AML Cells. Cancer Discov. *9*, 910–925.

14. Drusbosky, L.M., Vidva, R., Gera, S., Lakshminarayana, A.V., Shyamasundar, V.P., Agrawal, A.K., Talawdekar, A., Abbasi, T., Vali, S., Tognon, C.E., et al. (2019). Predicting response to BET inhibitors using computational modeling: A BEAT AML project study. Leuk. Res. *77*, 42–50.

15. Rosenberg, M.W., Watanabe-Smith, K., Tyner, J.W., Tognon, C.E., Druker, B.J., and Borate, U. (2020). Genomic markers of midostaurin drug sensitivity in FLT3 mutated and FLT3 wild-type acute myeloid leukemia patients. Oncotarget *11*, 2807–2818.

16. Kurtz, S.E., Eide, C.A., Kaempf, A., Long, N., Bottomly, D., Nikolova, O., Druker, B.J., McWeeney, S.K., Chang, B.H., Tyner, J.W., and Agarwal, A. (2022). Associating drug sensitivity with differentiation status identifies effective combinations for acute myeloid leukemia. Blood Adv. *6*, 3062–3067.

17. Kurtz, S.E., Eide, C.A., Kaempf, A., Khanna, V., Savage, S.L., Rofelty, A., English, I., Ho, H., Pandya, R., Bolosky, W.J., et al. (2017). Molecularly targeted drug combinations demonstrate selective effectiveness for myeloid- and lymphoid-derived hematologic malignancies. Proc. Natl. Acad. Sci. USA *114*, E7554–E7563.

18. Jayavelu, A.K., Wolf, S., Buettner, F., Alexe, G., Häupl, B., Comoglio, F., Schneider, C., Doebele, C., Fuhrmann, D.C., Wagner, S., et al. (2022). The proteogenomic subtypes of acute myeloid leukemia. Cancer Cell *40*, 301–317.e12.

19. Zeng, A.G.X., Bansal, S., Jin, L., Mitchell, A., Chen, W.C., Abbas, H.A., Chan-Seng-Yue, M., Voisin, V., van Galen, P., Tierens, A., et al. (2022). A cellular hierarchy framework for understanding heterogeneity and predicting drug response in acute myeloid leukemia. Nat. Med. *28*, 1212–1223.

20. Carey, A., Edwards, D.K., Eide, C.A., Newell, L., Traer, E., Medeiros, B.C., Pollyea, D.A., Deininger, M.W., Collins, R.H., Tyner, J.W., et al. (2017). Identification of Interleukin-1 by Functional Screening as a Key Mediator of Cellular Expansion and Disease Progression in Acute Myeloid Leukemia. Cell Rep. *18*, 3204–3218.

21. Traer, E., Martinez, J., Javidi-Sharifi, N., Agarwal, A., Dunlap, J., English, I., Kovacsovics, T., Tyner, J.W., Wong, M., and Druker, B.J. (2016). FGF2 from Marrow Microenvironment Promotes Resistance to FLT3 Inhibitors in Acute Myeloid Leukemia. Cancer Res. *76*, 6471–6482.

22. Edwards, D.K., Watanabe-Smith, K., Rofelty, A., Damnernsawad, A., Laderas, T., Lamble, A., Lind, E.F., Kaempf, A., Mori, M., Rosenberg, M., et al. (2019). CSF1R inhibitors exhibit antitumor activity in acute myeloid leukemia by blocking paracrine signals from support cells. Blood *133*, 588–599.

23. Joshi, S.K., Nechiporuk, T., Bottomly, D., Piehowski, P.D., Reisz, J.A., Pittsenbarger, J., Kaempf, A., Gosline, S.J.C., Wang, Y.-T., Hansen, J.R., et al. (2021). The AML microenvironment catalyzes a stepwise evolution to gilteritinib resistance. Cancer Cell *39*, 999–1014.e8.

24. Frejno, M., Meng, C., Ruprecht, B., Oellerich, T., Scheich, S., Kleigrewe, K., Drecoll, E., Samaras, P., Hogrebe, A., Helm, D., et al. (2020). Proteome activity landscapes of tumor cell lines determine drug responses. Nat. Commun. *11*, 3639.

25. Ali, M., Khan, S.A., Wennerberg, K., and Aittokallio, T. (2018). Global proteomics profiling improves drug sensitivity prediction: results from a multi-omics, pan-cancer modeling approach. Bioinformatics *34*, 1353–1362.

26. Rydenfelt, M., Wongchenko, M., Klinger, B., Yan, Y., and Blüthgen, N. (2019). The cancer cell proteome and transcriptome predicts sensitivity to targeted and cytotoxic drugs. Life Sci. Alliance *2*, e201900445.

27. Li, Y., Porta-Pardo, E., Tokheim, C., Bailey, M.H., Yaron, T.M., Stathias, V., Geffen, Y., Imbach, K.J., Cao, S., Anand, S., et al. (2023). Pan-cancer proteogenomics connects oncogenic drivers to functional states. Cell *186*, 3921–3944.e25.

28. Krug, K., Jaehnig, E.J., Satpathy, S., Blumenberg, L., Karpova, A., Anurag, M., Miles, G., Mertins, P., Geffen, Y., Tang, L.C., et al. (2020). Proteogenomic Landscape of Breast Cancer Tumorigenesis and Targeted Therapy. Cell *183*, 1436–1456.e31.

29. Hu, Y., Pan, J., Shah, P., Ao, M., Thomas, S.N., Liu, Y., Chen, L., Schnaubelt, M., Clark, D.J., Rodriguez, H., et al. (2020). Integrated Proteomic and Glycoproteomic Characterization of Human High-Grade Serous Ovarian Carcinoma. Cell Rep. *33*, 108276.

30. Clark, D.J., Dhanasekaran, S.M., Petralia, F., Pan, J., Song, X., Hu, Y., da Veiga Leprevost, F., Reva, B., Lih, T.-S.M., Chang, H.-Y., et al. (2019). Integrated Proteogenomic Characterization of Clear Cell Renal Cell Carcinoma. Cell *179*, 964–983.e31.

31. Huang, C., Chen, L., Savage, S.R., Eguez, R.V., Dou, Y., Li, Y., da Veiga Leprevost, F., Jaehnig, E.J., Lei, J.T., Wen, B., et al. (2021). Proteogenomic insights into the biology and treatment of HPV-negative head and neck squamous cell carcinoma. Cancer Cell *39*, 361–379.e16.

32. Dou, Y., Kawaler, E.A., Cui Zhou, D., Gritsenko, M.A., Huang, C., Blumenberg, L., Karpova, A., Petyuk, V.A., Savage, S.R., Satpathy, S., et al. (2020). Proteogenomic Characterization of Endometrial Carcinoma. Cell *180*, 729–748.e26.

33. Wang, L.-B., Karpova, A., Gritsenko, M.A., Kyle, J.E., Cao, S., Li, Y., Rykunov, D., Colaprico, A., Rothstein, J.H., Hong, R., et al. (2021). Proteogenomic and metabolomic characterization of human glioblastoma. Cancer Cell *39*, 509–528.e20.

34. Zhang, H., Liu, T., Zhang, Z., Payne, S.H., Zhang, B., McDermott, J.E., Zhou, J.-Y., Petyuk, V.A., Chen, L., Ray, D., et al. (2016). Integrated Proteogenomic Characterization of Human High-Grade Serous Ovarian Cancer. Cell *166*, 755–765.

35. McDermott, J.E., Arshad, O.A., Petyuk, V.A., Fu, Y., Gritsenko, M.A., Clauss, T.R., Moore, R.J., Schepmoes, A.A., Zhao, R., Monroe, M.E., et al. (2020). Proteogenomic Characterization of Ovarian HGSC Implicates Mitotic Kinases, Replication Stress in Observed Chromosomal Instability. Cell Rep. Med. *1*, 100004.

36. Koschade, S.E., Klann, K., Shaid, S., Vick, B., Stratmann, J.A., Thölken, M., Meyer, L.M., Nguyen, T.D., Campe, J., Moser, L.M., et al. (2022). Translatome proteomics identifies autophagy as a resistance mechanism to on-target FLT3 inhibitors in acute myeloid leukemia. Leukemia *36*, 2396–2407.

37. Ramsey, H.E., Stengel, K., Pino, J.C., Johnston, G., Childress, M., Gorska, A.E., Arrate, P.M., Fuller, L., Villaume, M., Fischer, M.A., et al. (2021). Selective Inhibition of JAK1 Primes STAT5-Driven Human Leukemia Cells for ATRA-Induced Differentiation. Targ Oncol *16*, 663–674.

38. Casado, P., Wilkes, E.H., Miraki-Moud, F., Hadi, M.M., Rio-Machin, A., Rajeeve, V., Pike, R., Iqbal, S., Marfa, S., Lea, N., et al. (2018). Proteomic and genomic integration identifies kinase and differentiation determinants of kinase inhibitor sensitivity in leukemia cells. Leukemia *32*, 1818–1822.

39. Hosseini, M.M., Kurtz, S.E., Abdelhamed, S., Mahmood, S., Davare, M.A., Kaempf, A., Elferich, J., McDermott, J.E., Liu, T., Payne, S.H., et al. (2018). Inhibition of interleukin-1 receptor-associated kinase-1 is a therapeutic strategy for acute myeloid leukemia subtypes. Leukemia *32*, 2374–2387.

40. Raffel, S., Klimmeck, D., Falcone, M., Demir, A., Pouya, A., Zeisberger, P., Lutz, C., Tinelli, M., Bischel, O., Bullinger, L., et al. (2020). Quantitative proteomics reveals specific metabolic features of acute myeloid leukemia stem cells. Blood *136*, 1507–1519.

41. Wang, J., Ma, Z., Carr, S.A., Mertins, P., Zhang, H., Zhang, Z., Chan, D.W., Ellis, M.J.C., Townsend, R.R., Smith, R.D., et al. (2017). Proteome Profiling Outperforms Transcriptome Profiling for Coexpression Based Gene Function Prediction. Mol. Cell. Proteomics *16*, 121–134.

42. Gosline, S.J.C., Tognon, C., Nestor, M., Joshi, S., Modak, R., Damnernsawad, A., Posso, C., Moon, J., Hansen, J.R., Hutchinson-Bunch, C., et al. (2022). Proteomic and phosphoproteomic measurements enhance ability to predict ex vivo drug response in AML. Clin. Proteomics *19*, 30.

43. Mertins, P., Tang, L.C., Krug, K., Clark, D.J., Gritsenko, M.A., Chen, L., Clauser, K.R., Clauss, T.R., Shah, P., Gillette, M.A., et al. (2018). Reproducible workflow for multiplexed deep-scale proteome and phosphoproteome analysis of tumor tissues by liquid chromatography–mass spectrometry. Nat. Protoc. *13*, 1632–1661.

44. Sanford, J.A., Wang, Y., Hansen, J.R., Gritsenko, M.A., Weitz, K.K., Sagendorf, T.J., Tognon, C.E., Petyuk, V.A., Qian, W.-J., Liu, T., et al. (2022). Evaluation of Differential Peptide Loading on Tandem Mass Tag-Based Proteomic and Phosphoproteomic Data Quality. J. Am. Soc. Mass Spectrom. *33*, 17–30.

45. Erba, H.P., Montesinos, P., Kim, H.-J., Patkowska, E., Vrhovac, R., Žák, P., Wang, P.-N., Mitov, T., Hanyok, J., Kamel, Y.M., et al. (2023). Quizartinib plus chemotherapy in newly diagnosed patients with FLT3-internal-tandem-duplication-positive acute myeloid leukaemia (QuANTUM-First): a randomised, double-blind, placebo-controlled, phase 3 trial. Lancet *401*, 1571–1583.

46. Brunet, J.-P., Tamayo, P., Golub, T.R., and Mesirov, J.P. (2004). Metagenes and molecular pattern discovery using matrix factorization. Proc. Natl. Acad. Sci. USA *101*, 4164–4169.

47. Friedman, J., Hastie, T., and Tibshirani, R. (2010). Regularization Paths for Generalized Linear Models via Coordinate Descent. J. Stat. Softw. *33*, 1–22.

48. McInnes, L., Healy, J., and Melville, J. (2020). UMAP: Uniform Manifold Approximation and Projection for Dimension Reduction. Preprint at arXiv.

49. Pino, J.C., Lubbock, A.L.R., Harris, L.A., Gutierrez, D.B., Farrow, M.A., Muszynski, N., Tsui, T., Sherrod, S.D., Norris, J.L., McLean, J.A., et al. (2022). Processes in DNA-damage response from a whole-cell multi-omics perspective. iScience *25*, 105341.

50. Gilbert, S.F. (2016). Developmental Plasticity and Developmental Symbiosis: The Return of Eco-Devo. Curr. Top. Dev. Biol. *116*, 415–433.

51. Wooten, D.J., and Quaranta, V. (2017). Mathematical models of cell phenotype regulation and reprogramming: Make cancer cells sensitive again. Biochim. Biophys. Acta. Rev. Cancer *1867*, 167–175.

52. Mbeunkui, F., and Johann, D.J. (2009). Cancer and the tumor microenvironment: a review of an essential relationship. Cancer Chemother. Pharmacol. *63*, 571–582.

53. Kramer, M.H., Zhang, Q., Sprung, R., Day, R.B., Erdmann-Gilmore, P., Li, Y., Xu, Z., Helton, N.M., George, D.R., Mi, Y., et al. (2022). Proteomic and phosphoproteomic landscapes of acute myeloid leukemia. Blood *140*, 1533–1548.

54. Aasebø, E., Berven, F.S., Bartaula-Brevik, S., Stokowy, T., Hovland, R., Vaudel, M., Døskeland, S.O., McCormack, E., Batth, T.S., Olsen, J.V., et al. (2020). Proteome and Phosphoproteome Changes Associated with Prognosis in Acute Myeloid Leukemia. Cancers *12*, 709.

55. Aasebø, E., Berven, F.S., Hovland, R., Døskeland, S.O., Bruserud, Ø., Selheim, F., and Hernandez-Valladares, M. (2020). The Progression of Acute Myeloid Leukemia from First Diagnosis to Chemoresistant Relapse: A Comparison of Proteomic and Phosphoproteomic Profiles. Cancers *12*, 1466.

56. Rifai, N., Gillette, M.A., and Carr, S.A. (2006). Protein biomarker discovery and validation: the long and uncertain path to clinical utility. Nat. Biotechnol. *24*, 971–983.

57. Röllig, C., Serve, H., Noppeney, R., Hanoun, M., Krug, U., Baldus, C.D., Brandts, C.H., Kunzmann, V., Einsele, H., Krämer, A., et al. (2021). Sorafenib or placebo in patients with newly diagnosed acute myeloid leukaemia: long-term follow-up of the randomized controlled SORAML trial. Leukemia *35*, 2517–2525.

58. Lee, M.J., Ye, A.S., Gardino, A.K., Heijink, A.M., Sorger, P.K., MacBeath, G., and Yaffe, M.B. (2012). Sequential Application of Anticancer Drugs Enhances Cell Death by Rewiring Apoptotic Signaling Networks. Cell *149*, 780–794.

59. Chipuk, J.E., Fisher, J.C., Dillon, C.P., Kriwacki, R.W., Kuwana, T., and Green, D.R. (2008). Mechanism of apoptosis induction by inhibition of the anti-apoptotic BCL-2 proteins. Proc. Natl. Acad. Sci. USA *105*, 20327–20332.

60. Bagci, E.Z., Vodovotz, Y., Billiar, T.R., Ermentrout, G.B., and Bahar, I. (2006). Bistability in Apoptosis: Roles of Bax, Bcl-2, and Mitochondrial Permeability Transition Pores. Biophys. J. *90*, 1546–1559.

61. Samra, B., Konopleva, M., Isidori, A., Daver, N., and DiNardo, C. (2020). Venetoclax-Based Combinations in Acute Myeloid Leukemia: Current Evidence and Future Directions. Front. Oncol. *10*, 562558.

62. Singh Mali, R., Zhang, Q., DeFilippis, R.A., Cavazos, A., Kuruvilla, V.M., Raman, J., Mody, V., Choo, E.F., Dail, M., Shah, N.P., et al. (2021). Venetoclax combines synergistically with FLT3 inhibition to effectively target leukemic cells in FLT3-ITD+ acute myeloid leukemia models. Haematologica *106*, 1034–1046.

63. Daver, N., Perl, A.E., Maly, J., Levis, M., Ritchie, E., Litzow, M., McCloskey, J., Smith, C.C., Schiller, G., Bradley, T., et al. (2022). Venetoclax Plus Gilteritinib for FLT3-Mutated Relapsed/Refractory Acute Myeloid Leukemia. J. Clin. Oncol. *40*, 4048–4059.

64. Raufaste-Cazavieille, V., Santiago, R., and Droit, A. (2022). Multi-omics analysis: Paving the path toward achieving precision medicine in cancer treatment and immuno-oncology. Front. Mol. Biosci. *9*, 962743.

65. Benard, B.A., Leak, L.B., Azizi, A., Thomas, D., Gentles, A.J., and Majeti, R. (2021). Clonal architecture predicts clinical outcomes and drug sensitivity in acute myeloid leukemia. Nat. Commun. *12*, 7244.

66. Maslah, N., Benajiba, L., Giraudier, S., Kiladjian, J.-J., and Cassinat, B. (2023). Clonal architecture evolution in Myeloproliferative Neoplasms: from a driver mutation to a complex heterogeneous mutational and phenotypic landscape. Leukemia *37*, 957–963.

67. Romer-Seibert, J.S., and Meyer, S.E. (2021). Genetic Heterogeneity and Clonal Evolution in Acute Myeloid Leukemia. Curr. Opin. Hematol. *28*, 64–70.

68. Schuringa, J.J., and Bonifer, C. (2020). Dissecting Clonal Heterogeneity in AML. Cancer Cell *38*, 782–784.

69. Sandén, C., Lilljebjörn, H., Orsmark Pietras, C., Henningsson, R., Saba, K.H., Landberg, N., Thorsson, H., von Palffy, S., Peña-Martinez, P., Högberg, C., et al. (2020). Clonal competition within complex evolutionary hierarchies shapes AML over time. Nat. Commun. *11*, 579.

70. Harris, L.A., Frick, P.L., Garbett, S.P., Hardeman, K.N., Paudel, B.B., Lopez, C.F., Quaranta, V., and Tyson, D.R. (2016). An unbiased metric of antiproliferative drug effect in vitro. Nat. Methods *13*, 497–500.

71. Hafner, M., Niepel, M., Chung, M., and Sorger, P.K. (2016). Growth rate inhibition metrics correct for confounders in measuring sensitivity to cancer drugs. Nat. Methods *13*, 521–527.

72. Langfelder, P., and Horvath, S. (2008). WGCNA: an R package for weighted correlation network analysis. BMC Bioinf. *9*, 559.

73. Langfelder, P., and Horvath, S. (2012). Fast R Functions for Robust Correlations and Hierarchical Clustering. J. Stat. Softw. *46*, i11.

74. Gaujoux, R., and Seoighe, C. (2010). A flexible R package for nonnegative matrix factorization. BMC Bioinf. *11*, 367.

75. Yu, G., Wang, L.-G., Han, Y., and He, Q.-Y. (2012). clusterProfiler: an R package for comparing biological themes among gene clusters. OMICS *16*, 284–287.

76. Ma, W., Kim, S., Chowdhury, S., Li, Z., Yang, M., Yoo, S., Petralia, F., Jacobsen, J., Li, J.J., Ge, X., et al. (2021). DreamAI: algorithm for the imputation of proteomics data. Preprint at bioRxiv.

77. Casado, P., Rodriguez-Prados, J.-C., Cosulich, S.C., Guichard, S., Vanhaesebroeck, B., Joel, S., and Cutillas, P.R. (2013). Kinase-substrate enrichment analysis provides insights into the heterogeneity of signaling pathway activation in leukemia cells. Sci. Signal. *6*, rs6.

78. igor (2022). Msigdbr: MSigDB Gene Sets for Multiple Organisms in a Tidy Data Format.

79. Liberzon, A., Birger, C., Thorvaldsdóttir, H., Ghandi, M., Mesirov, J.P., and Tamayo, P. (2015). The Molecular Signatures Database (MSigDB) hallmark gene set collection. Cell Syst. *1*, 417–425.

80. Ke, G., Meng, Q., Finley, T., Wang, T., Chen, W., Ma, W., Ye, Q., and Liu, T.-Y. (2017). LightGBM: A Highly Efficient Gradient Boosting Decision Tree. In Advances in Neural Information Processing Systems (Curran Associates, Inc.).

81. Monroe, M.E., Shaw, J.L., Daly, D.S., Adkins, J.N., and Smith, R.D. (2008). MASIC: a software program for fast quantitation and flexible visualization of chromatographic profiles from detected LC-MS(/MS) features. Comput. Biol. Chem. *32*, 215–217.

82. Gassaway, B.M., Li, J., Rad, R., Mintseris, J., Mohler, K., Levy, T., Aguiar, M., Beausoleil, S.A., Paulo, J.A., Rinehart, J., et al. (2022). A multi-purpose, regenerable, proteome-scale, human phosphoserine resource for phosphoproteomics. Nat. Methods *19*, 1371–1375.

83. Beausoleil, S.A., Villén, J., Gerber, S.A., Rush, J., and Gygi, S.P. (2006). A probability-based approach for high-throughput protein phosphorylation analysis and site localization. Nat. Biotechnol. *24*, 1285–1292.

84. Gillette, M.A., Satpathy, S., Cao, S., Dhanasekaran, S.M., Vasaikar, S.V., Krug, K., Petralia, F., Li, Y., Liang, W.-W., Reva, B., et al. (2020). Proteogenomic Characterization Reveals Therapeutic Vulnerabilities in Lung Adenocarcinoma. Cell *182*, 200–225.e35.

STAR★METHODS

KEY RESOURCES TABLE

REAGENT or RESOURCE	SOURCE	IDENTIFIER
Critical commercial assays		
BCA assay	ThermoFisher	23225
CellTiter96	AQueous One; Promega	G358C
Deposited data		
210 patient global proteomics	http://synapse.org/ptrc	syn25714248
210 patient phosphoproteomics	http://synapse.org/ptrc	syn32528196
210 proteomics	https://pdc.cancer.gov/pdc	PDC000477
Raw phosphoproteomics data	https://pdc.cancer.gov/pdc	PDC000478
159 patients RNAseq	Tyner et al.[11]	syn32529921
177 whole exome sequencing	Tyner et al.[11]	syn32533104
Ex Vivo drug response data	Tyner et al.[11]	syn25830473
Clinical Data	Tyner et al.[11]	syn26534982
Experimental models: Cell lines		
MOLM14	Dr. Yoshinobu Matsuo	N/A
Early and late quizartinib resistant MOLM14	Traer et al.[21]	N/A
Software and algorithms		
Analysis code	This paper	https://github.com/PNNL-CompBio/BeatAMLproteomics
MASIC	Maslah et al.[66]	N/A
WGCNA	Langfelder and Horvath[72,73]	N/A
NMF	Gaujoux and Seoighe[74]	N/A
clusterProfiler	Yu et al.[75]	N/A
DreamAI	Ma et al.[76]	N/A
glmnet	Friedman et al.[47]	N/A
KSEAapp	Casado et al.[77]	N/A
msigdbr	Igor,[78] Liberzon et al.[79]	N/A
MAGINE	Pino et al.[49]	N/A
lightGBM	Ke et al.[80]	N/A

RESOURCE AVAILABILITY

Lead contact

Further information and requests for resources and reagents should be directed to and will be fulfilled by the Lead Contact, Sara J.C. Gosline (sara.gosline@pnnl.gov).

Materials availability

This study did not generate new unique reagents.

Data and code availability

- All datasets generated from this study have been deposited onto Synapse and are available for public download. These can be accessed by navigating to http://synapse.org/ptrc to request data access for this study. Once access is provided, all files can be reached through their Synapse IDs provided in the key resources table, Proteomics data is also available at the proteomic data commons at https://pdc.cancer.gov/pdc/.
- All original code has been deposited at GitHub (https://github.com/PNNL-CompBio/BeatAMLproteomics) and is publicly available as of the date of publication. DOIs are listed in the key resources table.
- Any additional information required to reanalyze the data reported in this paper is available from the lead contact upon request.

EXPERIMENTAL MODEL AND STUDY PARTICIPANT DETAILS

Sample collection

We collected global and phosphoproteomics for 210 Beat AML patient samples for which we have genomic, transcriptomic, and *ex vivo* drug sensitivity data, summarized in Figure 1A. Samples were collected and processed as described in detail previously.[11,12] Briefly, all patients gave informed consent to participate in the Beat AML study, which had the approval and guidance of the Institutional Review Boards (IRB) from participating institutions. All samples used in this manuscript were processed at Oregon Health & Science University and had given explicit Informed Consent for additional analyses for research purposes. Mononuclear cells (MNCs) were isolated from freshly obtained bone marrow or peripheral blood samples from AML patients via Ficoll gradient centrifugation. No statistically significant differences in molecular analyses were evident on comparisons of bone marrow peripheral blood samples by Principal Components Analysis. Isolated MNCs were utilized for genomic (500x WES; RNA-seq) and *ex vivo* functional drug screens. WES and RNA-seq were performed using standard methods and data analysis was performed as previously described.[3] Clinical, prognostic, genetic, cytogenetic, and pathologic laboratory values as well as treatment and outcome data were manually curated from the patient electronic medical records (EMR). Patients were assigned a specific diagnosis based on the prioritization of genetic and clinical factors as determined by WHO guidelines. We selected 210 unique patients from the ongoing BeatAML study for complete proteomic and phosphoproteomic measurements (Table S1), based on the availability of sufficient material for proteomics and had some functional drug screens performed. The cohort was also developed to have sufficient power to analyze FLT3 ITD positive and FLT3 ITD negative patients, which required selecting more FLT3-ITD patients than occur in the general population. Some patients did not have enough sample volume for RNA-seq. Out of the 210 specimens, 122 were peripheral blood (58%), 80 bone marrow aspirate (38%), and 8 leukapheresis (4%). All clinical metadata was pulled from the previously published BeatAML study, including percentage of blast cells for each sample and other relevant data; importantly, due to changes in clinical practice over the course of the BeatAML study, FAB status was not annotated in the medical record for over 50% of the patients. All annotations can be found in the Clinical annotations spreadsheet on Synapse (syn25796769). A summary of sex, gender, age, drug treatment, response to induction therapy, and specimen type can be found in Table S6.

Quizartinib resistant cell line generation

Human MOLM14 cells were generously provided by Dr. Yoshinobu Matsuo (Fujisaki Cell Center, Hayashibara Biochemical Labs, Okayama, Japan). Cells were grown in RPMI (Life Technologies Inc., Carlsbad, CA) supplemented with 10% FBS (Atlanta Biologicals, Flowery Branch, GA), 2% L-glutamine, 1% penicillin/streptomycin (Life Technologies Inc.), and 0.1% amphotericin B (HyClone, South Logan, UT). Cell line authentication was performed at the OHSU DNA Services Core facility. To establish resistant cultures, 10 million MOLM14 cells were treated with 10 nM of quizartinib (Selleck Chemicals, Houston, TX) in media alone (N = 4) or in media supplemented with 10 ng/mL of FGF2 (N = 4) or FLT3 ligand (N = 4, FL; PeproTech Inc., Rocky Hill, NJ). All cultures were maintained in 10 mL of media. Every 2 or 3 days, recombinant ligands and quizartinib were replaced, and cell viability was evaluated using the Guava cell counter (Millipore Inc., Burlington, MA). Following ligand withdrawal, quizartinib and media were similarly replenished, and viability was monitored every 2 to 3 days. All cell lines were tested for mycoplasma on a monthly schedule. For proteomic and phosphoproteomic profiling, naive MOLM14 (N = 4), quizartinib-resistant parental (N = 2, no ligand), early (N = 4/ligand) and late (N = 4/ligand) cultures were washed three times with PBS to remove any trace of fetal bovine serum, pelleted, and flash frozen. For purposes of analyses, quizartinib-resistant parental and late cultures were combined with a final N of 6.

METHOD DETAILS

Ex vivo drug screening analysis

Drug sensitivity measurements were previously collected from the Beat AML[11,12] study. In this study, 10,000 viable cells were dispensed into each well of a 384-well plate containing 7-point, 3-fold dilution, drug concentration series from a library of small molecule inhibitors. Cells were incubated with the drugs in RPMI media containing 10% FBS without supplementary cytokines. After 3 days of culture at 37°C in 5% CO_2, MTS reagent (CellTiter96 AQueous One; Promega) was added, the optical density was measured at 490 nm, and raw absorbance values were adjusted to a reference blank value and then used to determine cell viability (normalized to untreated control wells). *Ex vivo* functional drug screen data processing was performed as described, and drug fitting was carried out using probit regression on quality-controlled data as in our previous work.[11,12]

Not all samples were exposed to every drug. To ensure sufficient samples to build the model, we selected drugs that were applied to at least 100 of the 210 patients. Additionally, we required at least 10 patients to exhibit drug sensitivity, which we defined as an AUC less than 100. We selected 46 drugs for analysis (Table S2).

Protein digestion and tandem mass tag (TMT) labeling

Sample preparation for proteomics was based on the protocols developed under the CPTAC consortium with minimal modifications.[28,43] For this study, all samples were confirmed to have TMT incorporation >98% using label incorporations tests described in.[43] Patient cell pellets were lysed with 500 μL fresh lysis buffer, containing 8 M urea (Sigma-Aldrich), 50 mM Tris pH 8.0, 75 mM

sodium chloride, 1 mM ethylenediamine tetra-acetic acid, 2 μg/mL Aprotinin (Sigma-Aldrich), 10 μg/mL Leupeptin (Roche), 1 mM PMSF in EtOH, 10 mM sodium fluoride, 1% of phosphatase inhibitor cocktail 2 and 3 (Sigma-Aldrich), 20 μM PUGNAc, and 0.01 U/μL Benzonase. The samples were then vortexed for 10 s and placed in a thermomixer for 15 min at 4°C and 800 RPM. Vortexing was repeated and the samples incubated again for 15 min utilizing the same settings. After incubation, the samples were centrifuged for 10 min at 4°C and 18,000 rcf to remove cell debris. The supernatant was then transferred to a fresh tube. BCA assay, 10-fold dilution, (ThermoFisher) was performed on the supernatant to determine protein yield.

Protein concentrations were normalized to the 2.0 μg/μL total protein prior to beginning digestion. The samples were reduced with 5 mM dithiothreitol (DTT) (Sigma-Aldrich) for 1 h at 37°C and 800 rpm. Reduced cystines were alkylated with 10 mM iodoacetamide (IAA) (Sigma-Aldrich) for 45 min at 25°C and 800 rpm in the dark. The sample was diluted 4-fold with 50 mM Tris HCL pH 8.0 and then Lys-C (Wako) was added at a 1:20 enzyme:substrate ratio, followed by incubation for 2 h at 25°C, shaking at 800 rpm. Trypsin (Promega) was then added at a 1:20 enzyme:substrate ratio, followed by a 14-h incubation at 25°C and 800 rpm. The sample was quenched by adding formic acid to 1% by volume and centrifuged for 15 min at 1500 rcf to remove any remaining cell debris. Peptides samples were desalted using a C18 solid phase extraction (SPE) cartridge (Waters Sep-Pak).

After drying down SPE eluates, each sample was reconstituted with 50 mM HEPES, pH 8.5 to a concentration of 5 μg/μL. Each isobaric tag aliquot was dissolved in 250 μL anhydrous acetonitrile to a final concentration of 20 μg/μL. The tag was added to the sample at a 1:1 peptide:label ratio and incubated for 1 h at 25°C and 400 rpm and then diluted to 2.5 mg/mL with 50 mM HEPES pH 8.5, 20% acetonitrile (ACN). Finally, the reaction was quenched with 5% hydroxylamine and incubated for 15 min at 25°C and 400 rpm. The samples were then combined per each plex set and concentrated in a speed-vac before a final C18 SPE cleanup. Each 11-plex experiment was fractionated into 96 fractions by high pH reversed phase separation, followed by concatenation into 24 fractions. Samples for global proteomics measurements were vialed at 0.1 μg/μL for LC-MS/MS analysis.

Phosphopeptide enrichment using IMAC

The global samples were further concatenated to create 12 samples per plex for further enrichment. Fe^{3+}-NTA-agarose beads were freshly prepared using Ni-NTA-agarose beads (Qiagen). Sample peptides were reconstituted to a 0.5 mg/mL concentration with 80% ACN, 0.1% trifluoroacetic acid (TFA) and incubated with 40 mL of the bead suspension for 30 min at RT in a thermomixer set at 800 rpm. After incubation the beads were washed with 100 mL 80% ACN, 0.1% TFA and 50 mL 1% formic acid (FA) to remove any non-specific binding. Phosphopeptides were eluted off beads with 210 mL 500 mM K_2HPO_4, pH 7.0 directly onto C18 stage tips and eluted from C18 material with 60 mL 50% ACN, 0.1% FA. Samples were dried in speed-vac concentrator for storage and reconstituted with 12 mL 3% ACN, 0.1% FA immediately prior to MS analysis.

LC-MS/MS analysis

Proteomic fractions were separated using a Waters nano-Aquity UPLC system (Waters) equipped with a 75 μm I.D. x 25 cm length C18 column packed in-house with 1.9 μm ReproSil-Pur 120 C18-AQ (Dr. Maisch GmbH). A 120-min gradient of 95% mobile phase A (0.1% (v/v) formic acid in water) to 19% mobile phase B (0.1% (v/v) FA in acetonitrile) was applied to each fraction. The separation was coupled to either a Thermo Orbitrap Fusion Lumos (patient samples) or Q Exactive HF (cell lines) Hybrid Quadrupole-Orbitrap mass spectrometer for MS/MS analysis. MS Spectra were collected from 350 to 1800 m/z at a mass resolution setting of 60,000. A top speed method was used for the collection of MS2 spectra at a mass resolution of 50K. An isolation window of 0.7 m/z was used for higher energy collision dissociation (HCD), singly charged species were excluded, and the dynamic exclusion window was 45 s. For the Fusion Lumos, a top speed method was used for the collection of MS2 spectra at a mass resolution of 50K. For the Q Exactive HF experiments, a top 16 method was used for the collection of MS^2 spectra at a mass resolution of 30K.

TMT global proteomics data processing

All Thermo RAW files were processed using mzRefinery to correct for mass calibration errors, and then spectra were searched with MS-GF + v9881[29–31] to match against the human reference protein sequence database downloaded in April of 2018 (71,599 proteins), combined with common contaminants (e.g., trypsin, keratin). A partially tryptic search was used with a ±10 parts per million (ppm) parent ion mass tolerance. A reversed sequence decoy database approach was used for false discovery rate calculation. MS-GF + considered static carbamidomethylation (+57.0215 Da) on Cys residues and TMT modification (+229.1629 Da) on the peptide N terminus and Lys residues, and dynamic oxidation (+15.9949 Da) on Met residues. The resulting peptide identifications were filtered to a 1% false discovery rate at the unique peptide level. A sequence coverage minimum of 6 per 1,000 amino acids was used to maintain a 1% FDR at the protein level after assembly by parsimonious inference.

The intensities of TMT-11 reporter ions were extracted using MASIC software.[81] Extracted intensities were then linked to peptide-spectrum matches (PSMs) passing the confidence thresholds described above. Relative protein abundance was calculated as the ratio of sample abundance to reference channel abundance, using the summed reporter ion intensities from peptides that could be uniquely mapped to a gene. The relative abundances were log2 transformed and zero-centered for each gene to obtain final relative abundance values. To facilitate robust comparisons, we filtered our dataset so that each feature is present in at least 50% of the samples, then the data was median polished. After filtering for at most 50% missingness, we obtain 8521 proteins.

TMT phosphoproteomics data processing

IMAC enriched fraction datasets were searched as described above with the addition of a dynamic phosphorylation (+79.9663 Da) modification on Ser, Thr, or Tyr residues. The phosphoproteomic data were further processed with the Ascore algorithm[82,83] for phosphorylation site localization, and the top-scoring assignments were reported. To account for sample loading biases in the phosphoproteome analysis, we applied the same sample correction factors derived from the median centering of the global proteomic dataset for normalization. We then filter the data so that each feature is present in at least 50% of the samples, and median polish the data. After filtering for at most 50% missingness, we obtain 18098 phosphosites. In MS-based phosphoproteomics, the occurrence of missing phosphosite data can be attributed to a multitude of factors. The high dynamic range of site abundance, sample complexity, patient-to-patient variability, technical variations in sample preparation batches, stochastic sampling of the MS during acquisition, the presence of other modifications affecting identification, and the lability of the phosphorylation modification. Additionally, stringent data processing criteria and biological variability introduce exclusions, resulting in missing values. The phosphosite missingness in our data is in line with other CPTAC human tumor studies[30,32,33,43]

Correcting proteomics and phosphoproteomics data for batch effect and loading mass

We filtered all proteins and phosphosites to reduce missingness by only selecting features that had at least one measurement in each of the 21 plexes. This yielded two datasets, with a total of 7084 proteins in the corrected global data (down from 8521), and 4082 phosphosites (down from 18098) in the corrected phospho data. Given the dramatic decrease in the number of phosphosites, and as a result, the low coverage of the FLT3 signaling network, we employed a more forgiving filter prior to correcting the processed phospho data: we allow phosphosites with at most 10 of the 21 plexes missing in the phospho data. This reduced our 18098 phosphosites down to 14084 phosphosites, a more reasonable drop. Further, there is very little change between the two corrections: the Pearson correlation between the two is greater than 0.972 across all features in common, indicating the more forgiving filter does a good job of including more phosphosites, while still leading to a dataset without confounding batch effects.

We plotted the samples by protein and phosphosite level (Figure S5A) and found that the peptide loading mass in each TMT channel correlates with the principal components (Figure S5B). This loading mass effect was also clearly detected using a t-test, where over 90% of features were significantly affected by loading mass (adjusted p value <0.05). The actual batch effect (the plex in which the sample ran) of the samples was more subtle, found in 14% of the global features and 56% of the phosphosites. To correct for both effects, we applied a Bayesian method implemented within the WGCNA[72,73] R package as the function empiricalBayesLM. After correction (Figure S5C), neither the effect due to loading mass nor plex is present. All proteomics data can be found on our Synapse site: http://synapse.org/ptrc. The cohort is spread across multiple files, as described in Table 1.

Multiomic clustering of patient subtypes via non-negative matrix factorization

We used the non-negative matrix factorization (NMF) R package[74] and the default NMF algorithm[46] to cluster the 159 patients for which we had transcriptomic, global proteomic, and phosphoproteomic measurements. NMF is a stochastic dimension reduction algorithm designed to approximate a given non-negative matrix X as a product of two smaller non-negative matrices, that is $X = WH,$ where the number of columns (rows) of $W(H)$ is determined by the user.

We adapted the data processing steps in Gillette[84] to our own data. Specifically, we imputed missing values from the global and phospho datasets using the KNN mode of DreamAI.[76] We then standardized every feature in each dataset to have mean zero and standard deviation equal to one. Each standardized feature then has both positive and negative values, so each of these is split into two: one piece containing only the positive expressions and the other the negative expressions, with zeros elsewhere. This yields a non-negative matrix (with twice as many rows as the original combined dataset) which we can factor using NMF as WH. The resulting non-negative matrix H (whose columns are samples) helps us determine clusters: we assign a sample to the cluster (row) with the maximum along the corresponding column of H. In a similar vein, the matrix W contains an aggregate expression for each feature (row) in each cluster (column); in applications the columns of W are commonly referred to as metagenes.

To determine the number k of clusters, we ran NMF a total of 50 times for each of the values $k = 2, -9$. The output of NMF depends on the starting conditions, so running 50 teams enables us to evaluate cluster stability. For each k we evaluate stability by inspecting the average adjacency matrix $A,$ which has samples in both the rows and columns, as well as by computing the cophenetic correlation (Figure S1B), which is a measure of how well the dendrogram obtained from A splits into k clusters. Representative results are shown in Figures S1C and S1D for various k values.

We decided not to use WES data for the 159 patients due to the sparsity of the mutational data: aside from FLT3/NPM1, the most common mutation only affected less than 25% of the patients and the 15th most common mutation affects less than 5%, with 601 mutations occurring in only a single patient. For comparison, proteomic, phospho-proteomic, and transcriptomic datasets had several thousand features each, with a wide range of values across the cohort.

Proteomic subtype predictor

We used non-negative matrix factorization (NMF) to classify samples into 4 subtypes with a combined proteomics, phosphoproteomics and transcriptomics dataset, classifying 159 of our 210 samples. However, as 51 of the samples lacked transcriptomic data, we

trained a multinomial regression model using the 159 NMF subtyped samples to extend the subtypes to all 210 samples, using the elastic net regularization, implemented in the R package glmnet.[47] Elastic net penalizes the coefficients using a combination of an L1 penalty (Lasso) and L2 penalty (Ridge), with the exact penalty given by $(1 - \alpha)\frac{||\beta||_2^2}{2} + \alpha||\beta||_1^2$, $0 \leq \alpha \leq 1$. For each $\alpha \in \{0, 0.1, \ldots 1\}$, we used stratified (subtypes split equally across splits), repeated, 5-fold cross validation to assess the recall, precision, and overall classification error of subtype predictor models trained using three datasets: proteomic, phospho-proteomic, and proteomic + phospho-proteomic data. In general, we found that the proteomic and phospho-proteomic models performed similarly well (Figure S3). Furthermore, while the choice of α greatly affected the number of non-zero features selected by Elastic Net, we found the effect of α on the classification errors, recall and precision was not nearly as pronounced, with the overall classification error ranging from 18.5% to 20% for all α values.

We found that subtype 3 was more difficult to recapture using proteomic and phospho-proteomic measurements in our models, specifically the recall of subtype 3 was quite variable across different α values. We chose $\alpha = 0.9$ to maximize the recall of subtype 3, as the model performance was otherwise comparable for different α values, as we can observe from the boxplots summarizing recall, precision, and overall error in Figures S3A and S3B.

With our choice of hyperparameters fixed, we trained a combined dataset of proteomic and phospho-proteomic data using all 159 NMF subtyped samples; we also manually inspected the 147 proteins + phospho-sites selected as relevant by this model and found clear expression patterns separating the patients by subtype (Figure 1B). We applied this model to all 210 patients, recapitulating the subtypes across the 159, and providing predictive subtype assignments to the 51 without transcriptomics.

We also employed this model to cell line data, by mean-centering and standardizing each feature, then imputing any missing values/features using the mean. While not a perfect solution, the model must see numerical data for all 147 of the features it uses, so imputing with the mean is a compromise when missing a few values or a few features. For each sample, the model outputs probabilities for the four subtypes adding up to one. The assigned subtype depicted in Figure 6 is the most likely according to the model.

Functional enrichment of subtypes

Toward further investigating the biological differences between the subtypes, we applied both KSEA and GSEA to the subtypes, using the mean within each subtype to rank all the genes. We used the clusterProfiler R package to calculate the GSEA results,[75] while the KSEAapp R package was used to compute KSEA.[77] The msigdbr R package[78,79] allowed us easy access to the Gene Ontology, Hallmarks, KEGG, Reactome and WikiPathways databases when applying GSEA.

Both GSEA and KSEA rely on a list of values corresponding to the aggregate activity within a subgroup of interest. We mean-center all the features, and for each subtype the average intensity is computed. This yields a named list of values ready for GSEA and KSEA. The raw p values from each enrichment test are adjusted using the Benjamini-Hochberg correction. We combined the enrichment results from each subtype to create the enrichment heatmaps shown in Figure 1 (which computes differences between clusters of 159 patients) and Figure 2 (which computes differences between clusters of 210 patients).

Prediction of drug-specific response

We expanded our previous modeling pipeline[42] with gradient boosted models using gradient boosted decision trees (GBT) using lightGBM.[80] Model features were composed of either phosphoproteomics, global proteomics, RNAseq, WES, or a combination of them all. We used the AUC of each drug for the target variable. Model parameters can be found in the online supplement notebook. For each drug, we performed repeated 5-fold cross validation a total of 5 times, resulting in 25 model training instances. This provided a way to estimate model reproducibility due to the small number of patient samples. First, we compared performance of Elastic Net (EN) and GBT (Figure S4A). We saw the GBT outperforms EN in nearly all cases (Figure S4B) and GBT can find dramatically less features yet provide better results (Figure S4A). We then compared impact of measurement choice. To easy represent our results, we now focus on the results for venetoclax, however we saw the same trend across all drugs. In agreement with our previous paper, we saw that global and phosphoproteomics provides similar performance to RNA alone or in combination (left panel Figure S4C). We also saw similar number of model features being selected across data type, regardless of the combination (middle panel of Figure S4C), there were significantly fewer biological enriched terms with features extracted from RNAseq (right panel of Figure S4C). We see that interoperability of features as important as the features themselves, thus focused on phosphoproteomics and global proteomics for further analysis. This was also supported by the fact that features selected when data types are using together showed the same features commonly being found regardless of model type (Figure S4D). After this cross-validation of the model, we then retrained the model across all available patient samples for the final feature selection model (used in Figure 4). For cell line testing, we retrained the model across 5-fold splits of the global and phosphoproteomics data, then tested the cell line data for each of the 5 models. Since there were multiple measurements per stage (naive, early, late), this resulted in a range of predictions, based on number of samples, times 5 models.

Signaling network construction

Drug centric signaling networks were constructed using MAGINEs Subnetwork class.[49] The background network was constructed using *generate_background_network*, leveraging Reactome_FI, KEGG, BioGrid, HMDB, and Signor, all available within the tool. Networks were constructed using the class function *paths_between_list*, which does a bidirectional search for shortest paths between species (BCL2/HDAC1 or MAP2K1/HDAC1).

QUANTIFICATION AND STATISTICAL ANALYSIS

Fishers exact test was used to test subtype enrichment of each clinical annotation and mutation status. P-value cutoff of 0.05 was used to determine significance. Drug response testing by mutation status or subtype was performed using Welch's t-test followed by FDR and BH corrections. The number of *s for each figure corresponds to the order of magnitude of significance (* = 0.01, ** = 0.001, etc).

Cell Reports Medicine, Volume *5*

Supplemental information

Mapping the proteogenomic landscape enables prediction of drug response in acute myeloid leukemia

James C. Pino, Camilo Posso, Sunil K. Joshi, Michael Nestor, Jamie Moon, Joshua R. Hansen, Chelsea Hutchinson-Bunch, Marina A. Gritsenko, Karl K. Weitz, Kevin Watanabe-Smith, Nicola Long, Jason E. McDermott, Brian J. Druker, Tao Liu, Jeffrey W. Tyner, Anupriya Agarwal, Elie Traer, Paul D. Piehowski, Cristina E. Tognon, Karin D. Rodland, and Sara J.C. Gosline

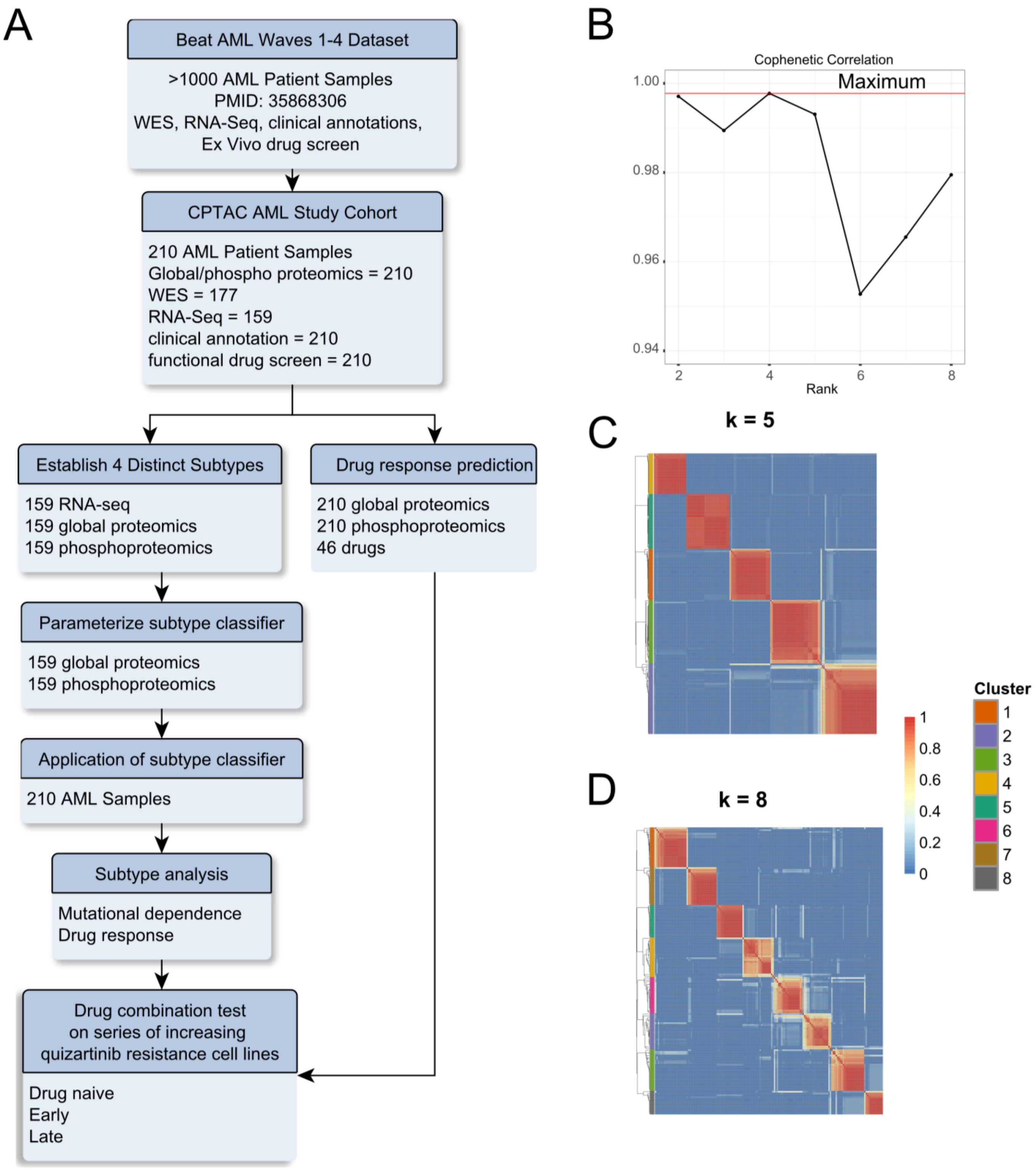

Figure S1. Patient cohort analysis. Related to Figure 1. (A) Flow of patient samples across modeling process. (B) The cophenetic correlation for each rank from NMF. For each rank k, this is a measure of the collective agreement between the clusters across 50 random initializations of NMF. Consensus heatmap of 50 NMF runs with k = 5 (C) and k = 8 (D) clusters. Ward method with Euclidian distance clustering was performed. The 159 samples are seen in the rows and columns, while the heatmap shows the proportion of runs in which samples were clustered together.

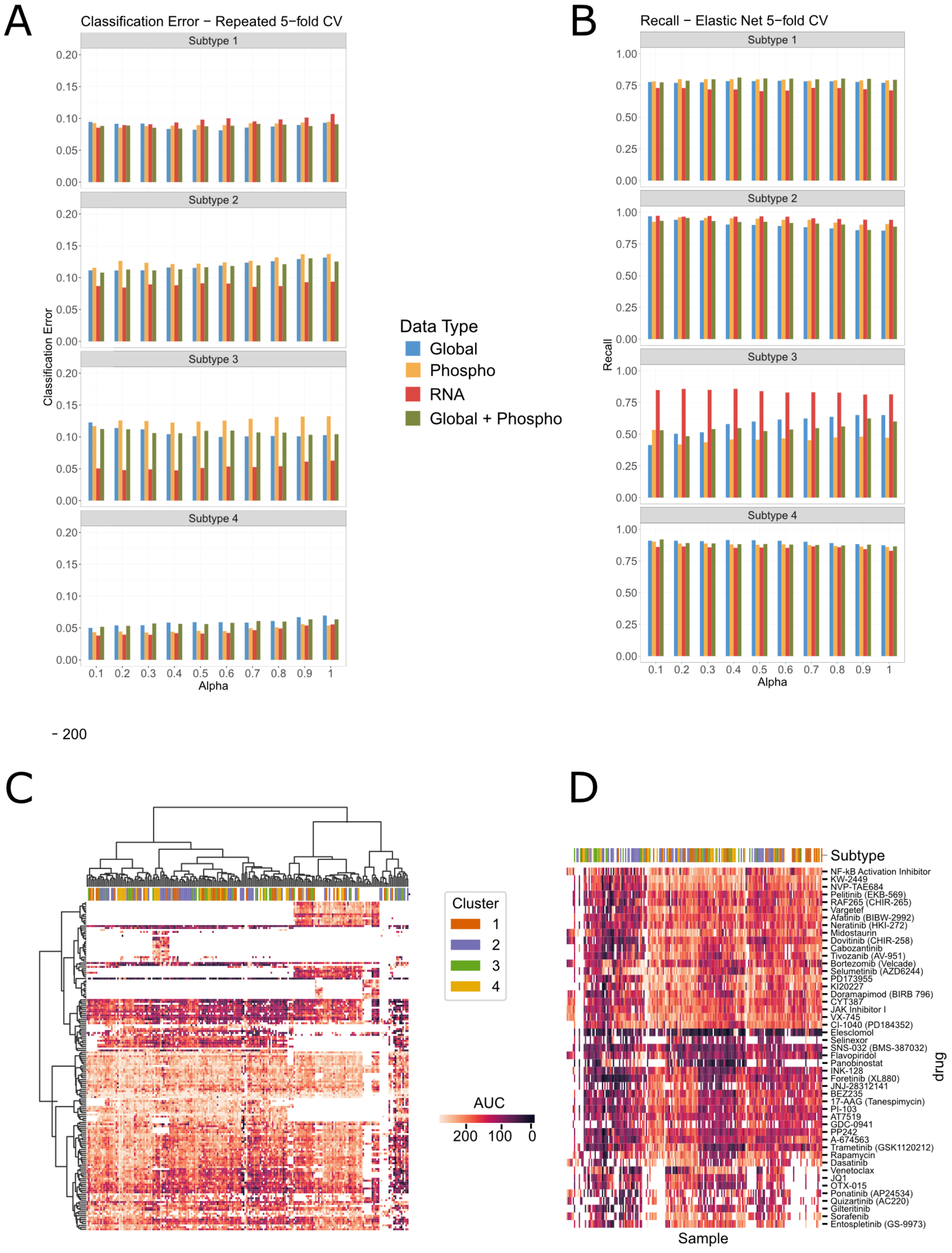

Figure S2. **Elastic net performance and extension.** Related to Figure 2. Classification error **(A)** and recall **(B)**, per subtype, for across values of α. **(C)** Heatmap of AUC values for available in the beatAML dataset **(D)** Resulting heatmap after filtering to include a minimum of 100 samples per drug.

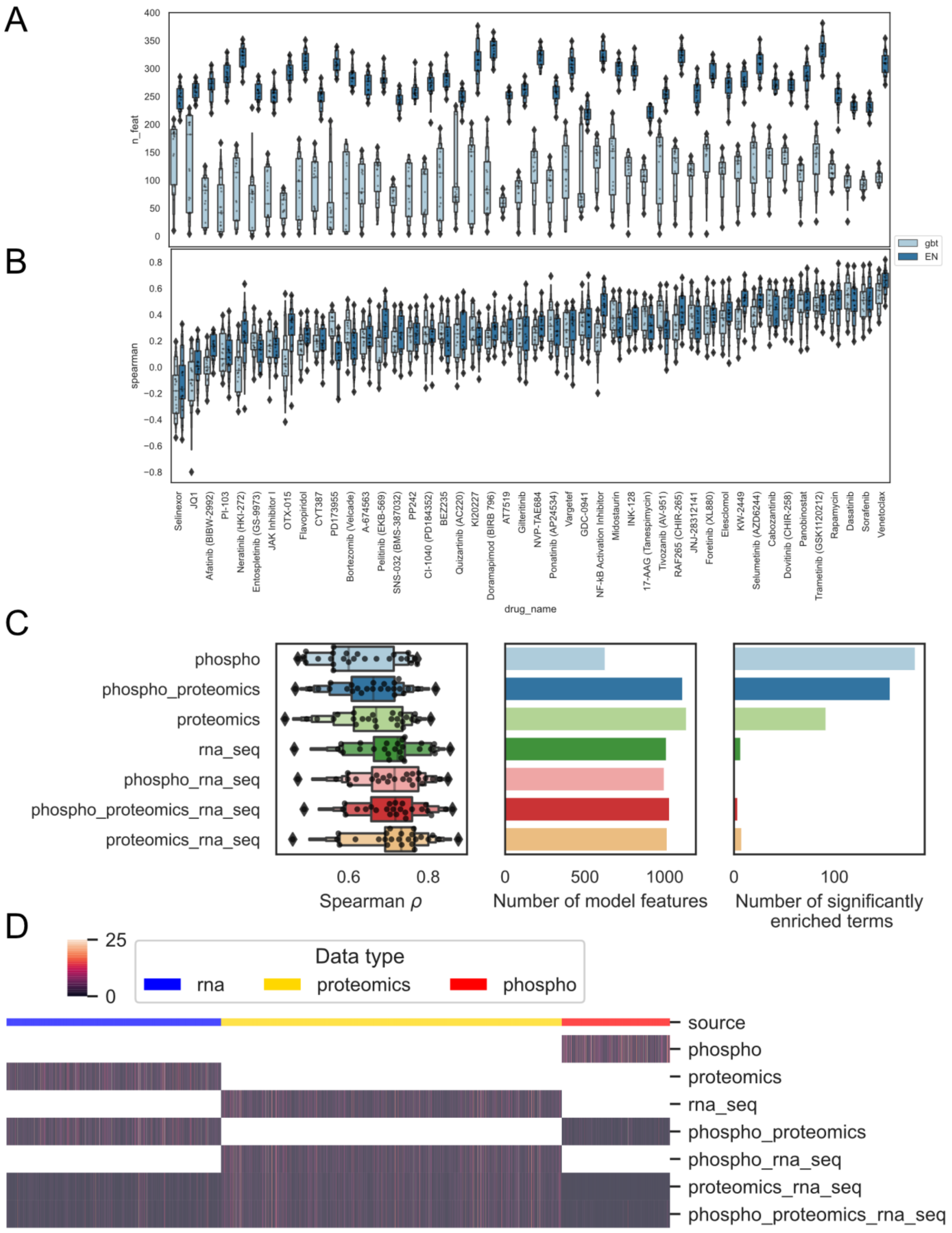

Figure S3. Predictive modeling of drug response using multi-omics. Related to STAR Methods. Number of features **(A)** and model performance **(B)** for Elastic Net (EN) or lightGBM (gbt). **(C)** Venetoclax performance for each data type and combinations of data type, as well as the number of features found, and the number of enriched terms for each data type. **(D)** Reoccurrence of model features for each data type. Since there were 5-repeat, 5-fold cross validation, a total of 25 models were constructed per data type. Thus, a feature could be selected 25 times. We see features are reselected across single and combination of data types, showing that additional data increases non-redundant information.

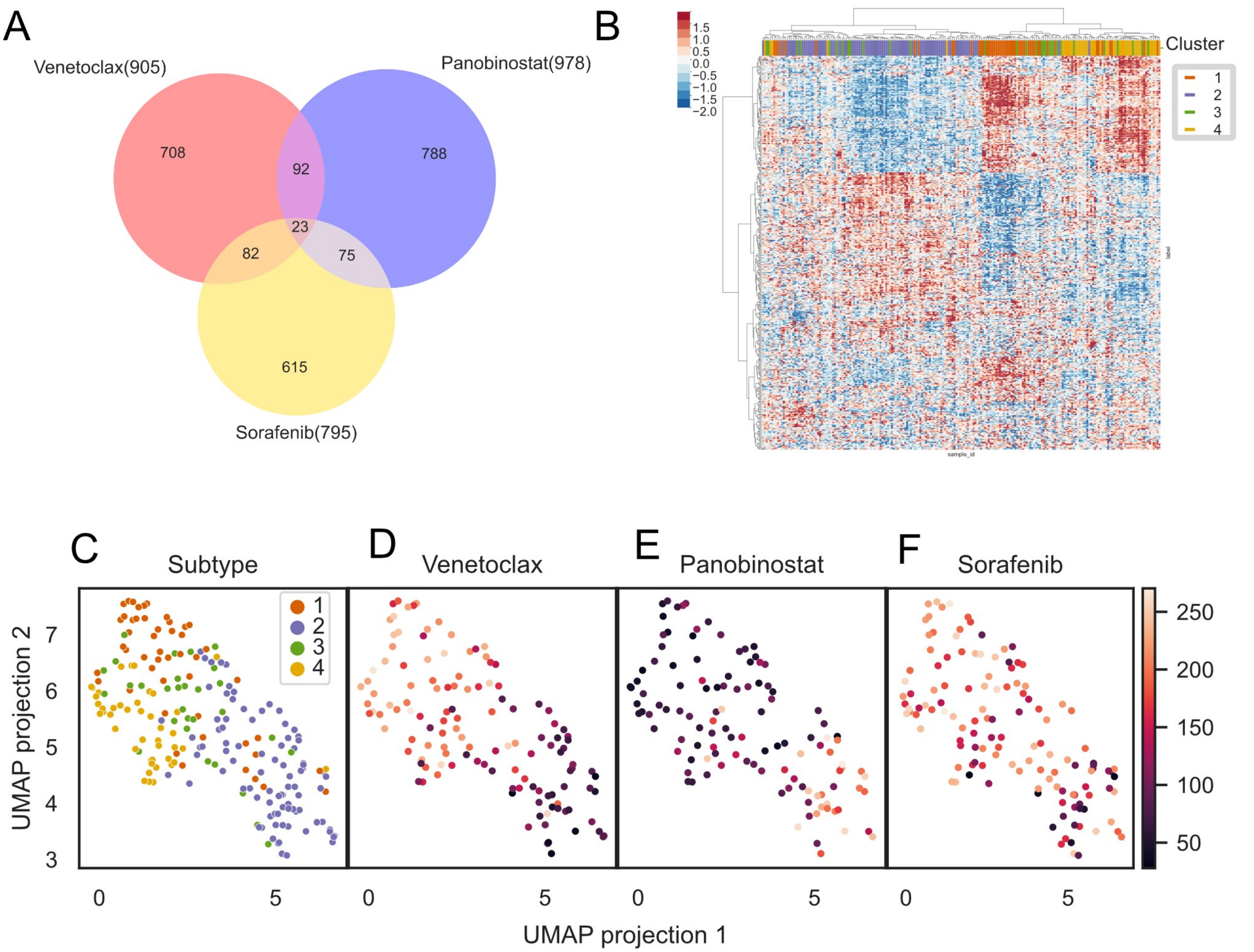

Figure S4. Feature comparisons between drugs of interest. Related to Figure 5. **(A)** Comparisons of features extracted using lightGBM. **(B)** Heatmap of the features from **(A)**, with unsupervised clustering. UMAP projection of the union of the features for each of the models, projected into two-dimensional landscape **(A-F)**. Color represented subtype **(C)** or AUC drug response (lower/darker values equates to more sensitive) **(D-E)**.

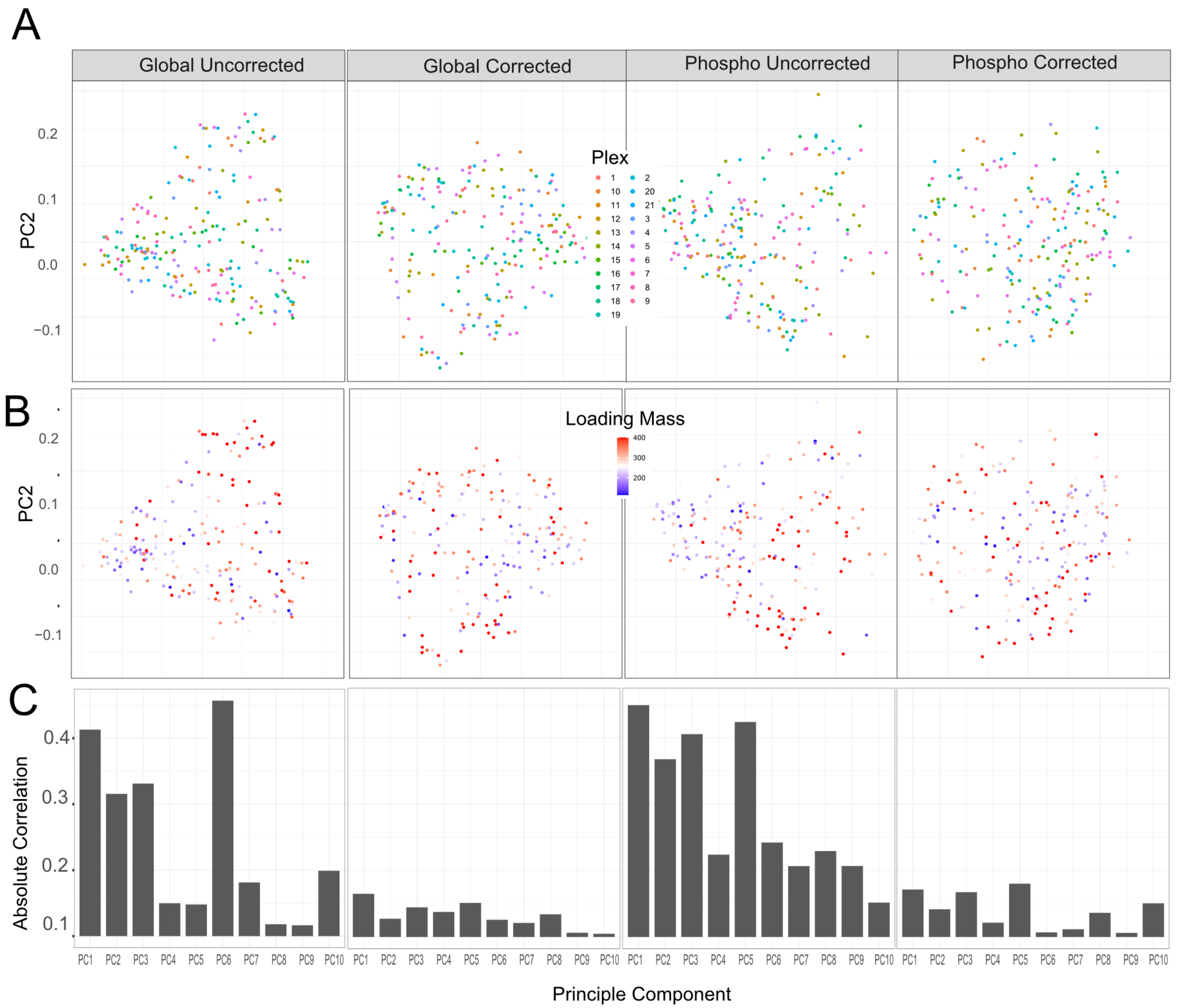

Figure S5. Batch correction of proteomics data. Related to STAR methods. First two principal components of Global and Phospho datasets before and after correction for Loading Mass **(A)** or Plex **(B)**. We found Loading Mass had a significant effect in 99% (global) and 93% (phospho) of the features while Plex had a significant effect in 14% (global) and 56% (phospho). After correction, neither Loading Mass nor plex no longer had any significant effect. The batch effect was assessed using BH-adjusted p-values from an F-test computed with the Limma package. **(C)** Correlation between Loading Mass and the first 10 principal components within Global and Phospho. Loading Mass has considerably higher correlation with the principal components in uncorrected data vs corrected data. After correction, little to no correlation is left between Loading Mass and the principal components.
